# Graphene Aerogel-Based Flexible Pressure Sensor for Physiological Signal Detection and Human–Machine Interaction

**DOI:** 10.1007/s40820-026-02109-8

**Published:** 2026-03-27

**Authors:** Zihan Wang, Zeshang Zhao, Qiyang Tu, Chengpeng Yao, Zhao Liu, Chengzhi Zhou, Luxiang Xu, Shijie Guo, Chuizhou Meng, Gaofeng Shao, Huanyu Cheng, Li Yang

**Affiliations:** 1 State Key Laboratory for Reliability and Intelligence of Electrical Equipment, Hebei Key Laboratory of Smart Sensing and Human-Robot Interaction, School of Mechanical Engineering, Hebei University of Technology, Tianjin, 300401 People’s Republic of China; 2 State Key Laboratory of Reliability and Intelligence of Electrical Equipment, School of Health Sciences and Biomedical Engineering, Hebei University of Technology, Tianjin, 300130 People’s Republic of China; 3 Research Institute of Wearable Electronic Materials and Devices, School of Materials Science and Engineering, Zhejiang Sci-Tech University, Hangzhou, 310018 People’s Republic of China; 4 School of Chemistry and Materials Science, Nanjing University of Information Science and Technology, Nanjing, 210044 People’s Republic of China; 5 Department of Engineering Science and Mechanics, The Pennsylvania State University, University Park, 16802 USA

**Keywords:** Piezoresistive pressure sensor, Graphene aerogel, Teleoperation, Force feedback, Intelligent object recognition

## Abstract

Despite extensive development of flexible pressure sensors, it is still difficult for them to simultaneously achieve high precision and a large response to subtle pressures. To address these challenges, this work demonstrates a flexible pressure sensing platform that features the reduced graphene oxide aerogel sandwiched between a polydimethylsiloxane encapsulation layer and a thin polyimide film with interdigital electrodes. The resulting pressure sensor exhibits a high sensitivity of 698.96 kPa^−1^ and a low limit of detection (~ 1 Pa), and outstanding stability over 20,000 loading/unloading cycles. Besides monitoring various physiological signals and human motions, the flexible pressure sensors can be configured into an array layout as a smart artificial electronic skin to recognize the spatial pressure distribution. The flexible pressure sensor can also be integrated with signal processing and wireless communication modules as a teleoperation system for gesture recognition, force feedback control, and kitchen food recognition, highlighting future potential toward smart robotics and human–machine interfaces.

**Supplementary Information:**

The online version contains supplementary material available at 10.1007/s40820-026-02109-8.

## Highlights

- Reduced graphene oxide aerogel (rGOA) was prepared via freeze-casting, featuring an ultra-light density (10 mg cm^−3^) and a unique anisotropic structure, which bring advantages to pressure sensing.
- The rGOA-based pressure sensor exhibits a sensitivity as high as 698.96 kPa^−1^, a detection range as wide as 100 kPa, and a cyclic stability of over 20,000 cycles.
- The integration of rGOA with manipulators enables teleoperation, stable grasping of fragile objects with force-feedback and 100% accuracy in food recognition.

**Supplementary Information:**

The online version contains supplementary material available at 10.1007/s40820-026-02109-8.

## Graphical abstract

**Figure:**
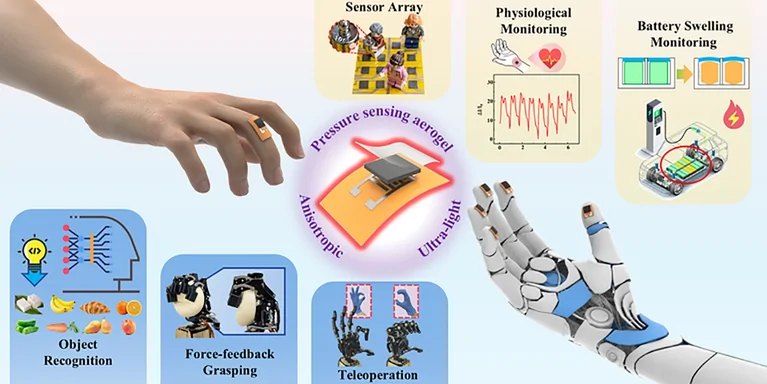

## Introduction

Flexible sensors based on piezoelectric [1, 2], capacitive [3–5], triboelectric [6–9], magnetoelectronics [10, 11] and piezoresistive [12–14] working mechanisms have been widely applied for human physiological signal monitoring [15–18], medical treatment [19–22], electronic skin [23–26], human–machine interfaces [27–31], and intelligent robotics [32, 33]. Among them, piezoresistive sensors have attracted considerable attention due to easy fabrication, fast response, and convenient signal acquisition. Commonly used piezoresistive sensing materials include MXene [34–36], graphene [37, 38], metal nanoparticles/nanowires [39–41], and carbon nanotubes [42, 43]. In particular, 3D-structured graphene aerogel with a conductive network of 2D graphene nanosheets further exhibits elastic and lightweight properties, the high electron mobility and conductivity of graphene provide the sensors with high sensitivity and fast response. Methods to prepare 3D graphene aerogels include self-assembly [44, 45], template method [46, 47], 3D printing [48, 49], and chemical reduction [50, 51]. As the mechanical properties of graphene aerogels hinge on their microstructures (e.g., honeycomb [52–54], lamellar [55, 56], and sphere-shaped [57, 58]), a large degree of deformation upon external pressure and thus a high sensitivity can be modulated and achieved.

Anisotropic aerogels with regular, single-direction-grown pores inside the structure exhibit elasticity and compression recoverability along the radial direction, providing sufficient deformation space and a larger pressure sensing range [59, 60]. Traditional sacrificial template methods usually involve acid etching or high-temperature carbonization, resulting in structural damage, poor order, and challenges to precisely control the pore architecture. 3D printing has strict requirements on the viscoelasticity and fluidity of the raw materials, along with the high equipment cost, to limit its applicability. In contrast, stable anisotropic structures can be generated by directional growth of ice crystals, followed by freeze-drying to fabricate aerogels with anisotropic pore structures. Freeze-casting is applicable to a wide range of material systems (nanoparticles, nanotubes, nanowires, nanosheets, polymer chains, and macromolecules), and the microstructure of the resulting aerogels, including porosity and pore morphology (layered, honeycomb, and radial), can be tailored by adjusting processing conditions, offering significant advantages [61–64].

This study employs freeze-casting to fabricate an ultralight and anisotropic reduced graphene oxide aerogel (rGOA). This rGOA is then integrated as the sensing element within a flexible pressure sensor by sandwiching it between a polyimide (PI) film coated with interdigital electrodes and a thin polydimethylsiloxane (PDMS) encapsulation layer. Due to the anisotropic structure of rGOA, the resulting pressure sensor exhibits a high sensitivity of up to 698.96 kPa^−1^, a broad range of detection to 100 kPa, a limit of detection of 1 Pa, and excellent stability over 20,000 loading/unloading cycles. In addition, the rGOA-based pressure sensors with ultra-high sensitivity have been explored to accurately detect varying physiological signals and human motions. Configuring the pressure sensors into an array layout can detect the pressure distribution and dynamic changes of multiple objects. The manipulator teleoperation system with signal processing and wireless communication modules further allows for gesture and kitchen food recognition, along with stable object grasping with force feedback to avoid object damage.

## Experimental Section

### Preparation of Graphene Aerogel

Sodium alginate (SA) solution (20 mg mL^−1^) was prepared by dissolving SA powder in deionized water at 90 °C. Graphene oxide (GO) suspension (10 mg mL^−1^) was prepared by dispersing GO powder in deionized water. A SA-GO suspension was then formed by mixing 9 mL of the GO suspension with 2.5 mL of the SA solution, followed by stirring at 500 rpm for 1 h to ensure homogeneity. SA-GO aerogels were fabricated using a unidirectional freezing method in a custom freeze-casting apparatus. The prepared SA-GO suspension was poured into an acrylic mold (10 mm × 10 mm × 5 mm) placed on a copper cold plate. After complete freezing at a controlled cooling rate of 8 °C min^−1^, the samples were freeze-dried for 72 h. Finally, rGO aerogels were obtained by thermally annealing the SA-GO aerogels at 800 °C for 1 h under an argon atmosphere.

### Preparation of the Pressure Sensor

The interdigital electrodes were prepared by screen printing (200 mesh) and drying of silver paste (85 °C, 30 min) on the PI thin film with a thickness of 35 μm (Dongxuan, Jiangsu, China). Next, the PDMS solution (Sylgard 184, Sigma-Aldrich, USA) with 1 g of prepolymer and 0.1 g of crosslinking agent in a mass ratio of 10:1 was applied on the PI film attached to a glass slide, followed by curing in a vacuum drying oven (100 °C, 60 min). Sandwiching the rGOA film (10 mm × 10 mm × 0.6 mm) between the interdigitated electrodes on PI (effective contact area of 1 cm^2^) and the PDMS encapsulation layer resulted in the rGOA-based pressure sensor. The connection between the electrodes and the data acquisition system was made by two copper foils (thickness of 0.05 mm) with silver paste (Ausbond). The PDMS precursor was cast into a laboratory spoon mold and thermally cured to obtain a fingertip-pulp-shaped PDMS sample (25 mm × 15 mm × 3 mm). In the battery swelling simulation, the pressure sensor array was inverted and placed on the battery casing, where the sensing layer captured the pressure signals induced by balloon expansion, thereby simulating and monitoring battery swelling.

### Characterization

The microstructure of rGOA was characterized using scanning electron microscope (SEM; Gemini SEM 500, Carl Zeiss, Germany). X-ray diffraction (XRD) patterns were acquired using a Rigaku D/teX Ultra 250 detector with Cu Kα radiation (40 kV, 40 mA). Raman spectroscopy was performed on a HORIBA HR800 spectrometer (532 nm excitation wavelength). X-ray photoelectron spectroscopy (XPS) analysis was conducted on an ESCALAB250Xi instrument (Thermo Fisher Scientific).

The external pressure was applied by a universal materials testing machine (JSV-H1000). The current output of the rGOA-based pressure sensor was measured by a Keithley 2400 source meter at 0.1 V. The pressure distribution from the 4 × 4 pressure sensor array was detected in real-time by a microcontroller (Arduino Mega 2560) connected to a computer using a custom-built MATLAB (Math Works) program.

The electrochemical impedance spectroscopy (EIS) of the rGOA was measured by an electrochemical workstation (Vertex.C.EIS), with the rGOA as the working electrode, a platinum sheet as the counter electrode, and a saturated calomel electrode as the reference. The measurements were conducted in a phosphate buffer solution (0.01 mol/L) over a frequency range from 100,000 to 0.1 Hz, with a DC potential of 0 V and an AC amplitude of 5 mV.

## Results and Discussion

### Design and Overview of the rGOA-based Pressure Sensor

**Fig. 1:**
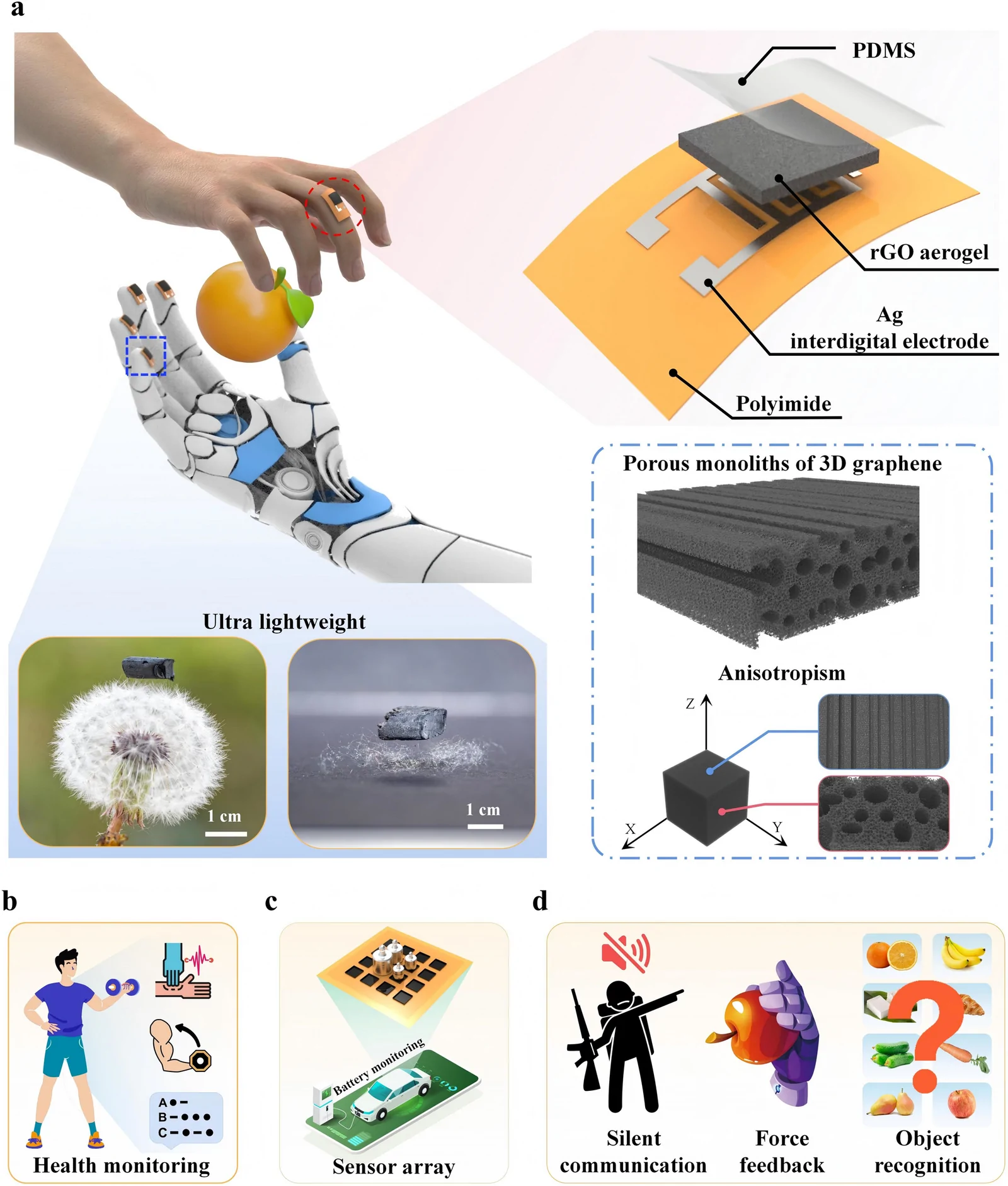
**a** Schematic showing the rGOA-based pressure sensor consisting of the interdigital electrodes, anisotropic and ultralight rGO aerogel, and PDMS encapsulation in sandwiched structure. rGOA-based pressure sensors can be applied for **b** monitoring physiological signals, **c** configured in an array layout to detect the spatial pressure distribution, and **d** on fingers for teleoperation, manipulation with force feedback, and object recognition

The flexible pressure sensor is designed by sandwiching rGOA between the screen-printed silver interdigitated electrodes on the PI substrate and a thin PDMS encapsulation layer (Fig. S1). The ultralight rGOA (with a density of 0.01 g cm^−3^) also exhibits anisotropic structures and properties, with tubular pores along one direction (Figs. 1 a and S2). The unique interconnected network of the lamellar rGOA can effectively dissipate the mechanical energy upon pressure [65, 66] to increase the upper limit of detection to 100 kPa. Meanwhile, the support along the Z-axis provided by the lamellar rGO can result in a rapid recovery time (40 ms) and outstanding cyclic stability (> 20,000 cycles at 10 kPa). Besides precise monitoring of physiological signals such as human body movements and pulse (Fig. 1b), the rGOA-based pressure sensors can also be arranged into an array layout and integrated with the Arduino development kit to achieve spatial pressure mapping and dynamic display (Fig. 1c). It can also be attached to a robotic manipulator to achieve teleoperation, force feedback, and intelligent object recognition for human*–*machine interactions (Fig. 1d).

### Material Characterization of the rGOA

**Fig. 2:**
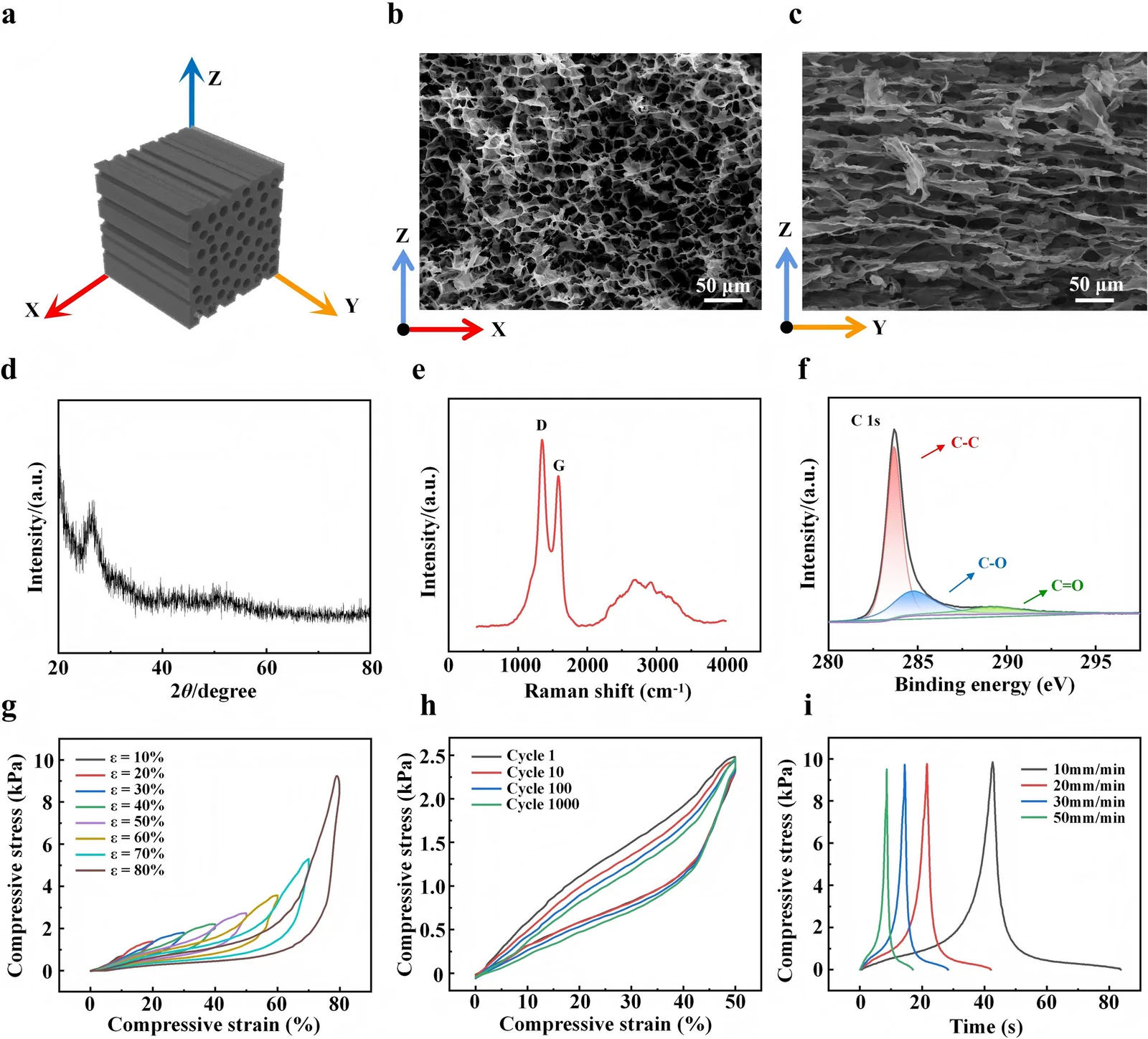
Characterization of the rGOA. **a** Schematic and **b** SEM images of the rGOA to show the porous structure along the Y-axis and **c** tubular structure along the X-axis. **d** XRD, **e** Raman, and **f** the deconvoluted C 1*s* spectrum of rGOA. **g** Compressive stress–strain curves of the rGOA under the applied strain from 10% to 80%. **h** Cycling stability of the rGOA measured during 1000 cycles at a strain of 50%. **i** Comparison of the compressive stress curves of the rGOA at different compression rates

The rGOA with anisotropic structure (Fig. 2a) features a “honeycomb-like” porous structure along the Y-axis (Fig. 2b) and a neatly arranged tubular structure along the X-axis (Fig. 2c). The anisotropic structure of rGOA originates from the unidirectional freezing, which leads to the unidirectional growth of ice crystals and the tubular pores during freeze-drying. The (002) diffraction peak at 2θ = 26.5° in the X-ray diffraction (XRD) (Fig. 2d) suggests the successful transformation of SA-GO aerogel into rGO aerogel. The presence of two prominent peaks at approximately 1348 cm^−1^ (D peak for disordered carbon) and 1582 cm^−1^ (G peak for graphite crystal) in Raman spectroscopy, with an I_D_/I_G_ ratio of 1.22, confirms the presence of reduced graphene oxide (Fig. 2e). The deconvoluted C 1*s* with peaks at 283.7 (C–C), 284.2 (C–O), and 289.3 eV (C = O) indicates the partial reduction of graphene oxide (Fig. 2f).

With the pressure testing platform (Fig. S3), the rGOA exhibits increased stress as the strain (along the Z-axis) increases from 10% to 80% and complete recovery over loading–unloading cycles (Fig. 2g). The porous framework grown along the Y-axis mainly supports compression along the Z-direction and is not intrinsically stretchable. Consequently, the rGOA collapses at 60% compressive strain, and loses most support at 70%-80% strain, leading to a sharp rise in the curve from material stacking (Fig. S4). The hysteresis in the stress–strain curve originates from the irreversible energy dissipation of rGOA during the loading–unloading process. Loading causes the elastic deformation of rGOA (recoverable) and molecular chain friction (irrecoverable). During unloading, friction between molecular chains continues to dissipate energy, causing the strain recovery rate to lag behind the stress release rate [67, 68]. The measured stress retention rate of 96.9% after 1,000 cycles under 50% compressive strain (Fig. 2h) or 82.6% after 100 cycles under 80% strain (Fig. S5) showcases excellent anti-fatigue properties of the rGOA*.* The effect of the compression rate ranging from 10 to 50 mm min^−1^ on the maximum stress amplitude is negligibly small (< 3%) (Fig. 2i), indicating rate-independent compression performance for stable cyclic operation under large strains.

### Sensing Properties of the rGOA-based Pressure Sensor

The sensing performance of the rectangular interdigital electrode depends on the finger width (W), spacing (S), and length (L) (Fig. S6). After first fixing L to 7.4 mm (to determine the overall size) and S to 0.6 mm, varying W (e.g., 1, 0.8, and 0.6 mm) investigates the effect of the aspect ratio. After selecting the width W of 0.8 mm, the spacing S is varied (e.g., 0.8, 0.6, and 0.4 mm) (Figs. S7 and S8). Finally, design optimization of the interdigital electrode results in the choice of an interdigital length of 7.4 mm, width of 0.8 mm, and finger spacing of 0.6 mm for enhanced current responses under a pressure range from 0 to 10 kPa. The pore structure of rGOA strongly influences its sensing performance. As the cooling rate increases from 2 to 8 and 15 °C min^*−*1^, the average pore size of the rGOA decreases from 22 to 15 and 10 μm, respectively (Fig. S9). This is because slower cooling allows sufficient time for ice crystal nuclei to grow. Since the vertical channels inherit the shape and size of the ice crystals, larger cooling times result in larger pore sizes. The current response decreases with increasing cooling rate because denser pores provide more initial conductive pathways, resulting in fewer pathway changes under compression and thus lower sensitivity. Although the 2 °C min^−1^ sample shows the highest current change, its large pores reduce the modulus and recovery ability, causing a rapid baseline drift; thus, the cooling rate of 8 °C min^*−*1^ is selected for further studies (Fig. S10). As a structural reinforcing and binding agent, SA strengthens the interlayer crosslinking of GO sheets through hydrogen bonding and electrostatic interactions, thereby significantly enhancing the mechanical integrity and stability of the aerogel. However, excessive SA reduces conductive pathways and current variation. Thus, an SA:GO ratio of 1:3 is chosen (Fig. S11). The comparison in the sensor performance with different thicknesses (50, 100, 150, and 200 μm) for the PDMS encapsulation indicates a negligibly small impact on the sensing performance (Figs. S12 and S13). However, the sensor with a thinner encapsulation can detect a smaller minimum pressure of 1 Pa (Fig. S14). Therefore, the PDMS encapsulation with a thickness of 50 μm is selected in the following study unless specified otherwise (Fig. S15). The PDMS encapsulation allows the rGOA-based sensor to maintain a stable current response under varying relative humidity (RH) levels from 20% to 80% (Fig. S16) for use in real-world environments. As the resistance (*R*_c_) of the silver interdigital electrodes remains unchanged upon pressure loading, the resistance (*R*_a_) of the rGOA itself and the contact resistance (*R*_b_) between the rGOA and the interdigitated electrodes decrease due to increased conductive pathways (Figs. S17 and S18), as directly reflected by the gradually increased LED brightness (Fig. S19).

**Fig. 3:**
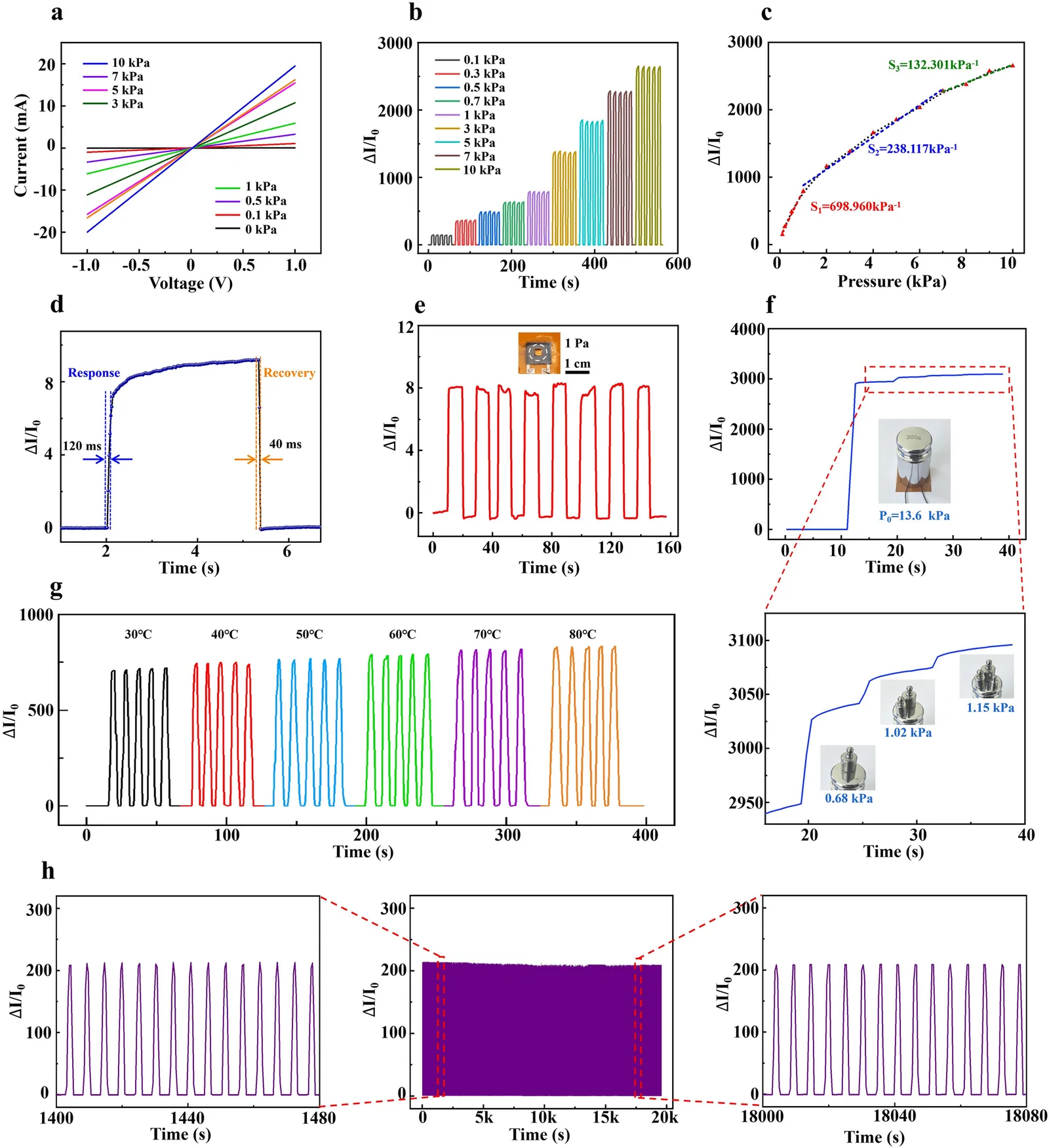
Electromechanical characterization of the flexible rGOA-based pressure sensor. **a** I − V curves and **b** normalized relative current changes of the rGOA-based pressure sensor under varying pressure loads from 0.1 to 10 kPa, along with **c** the calibration curve to determine the sensitivity. **d** Response and recovery time to the applied pressure of 1 Pa. **e** Current response to 1 Pa over eight cycles. **f** Detection of additional small pressure (0.68, 1.02, and 1.15 kPa from the weight of 10, 15, and 16 g) under an existing high pressure of 13.6 kPa. **g** Current response of the rGOA-based pressure sensor at a range of 30–80 °C. **h** Repeatability test of the rGOA-based sensor over 5000 loading/unloading cycles to a pressure of 250 Pa

Benefiting from anisotropic characteristics, the porous structure of rGOA provides abundant compressible space that generates new conductive pathways under pressure loading, thereby enhancing detection limit and sensitivity. Meanwhile, the tubular walls offer structural support, preventing collapse under large or repeated loads and enabling rapid recovery of the porous network upon unloading [65, 66]. Compared with the isotropic structure, the anisotropic structure exhibits lower peak stress and a more uniform stress and strain distribution, as revealed in the COMSOL simulations with the same applied load (Fig. S20), indicating more effective load transfer, reduced stress concentration, and a more stable evolution of conductive pathways under pressure loading. The linear current*–*voltage (I-V) curves of the rGOA-based pressure sensor in response to the pressure ranging from 0 to 100 kPa (Figs. 3a and S21a) indicate stable ohmic contact over the large pressure range. The sensor also exhibits a highly repeatable and stable response to the pressure from 0.1 to 100 kPa (Figs. 3b and S21b), over the frequency range from 0.03 to 0.1 Hz (5 kPa) and the compression rate from 5 to 50 mm min^*−*1^ (Fig. S22). Although the normalized relative current output changes nonlinearly with the applied pressure, it can be fitted piecewise linearly with a sensitivity of 698.96, 238.12, 132.30, 11.40, and 3.25 kPa^−1^ in the pressure ranges of 0.1–1, 1–7, 7–10, 10–30, and 30–100 kPa, respectively (Figs. 3c and S23). The segmented sensitivity of the sensor arises from the differences in the deformation mechanisms and mechanical properties of rGOA under increased pressures. In the low-pressure range, the internal pores of rGOA compress rapidly, forming numerous conductive pathways that cause a significant decrease in resistance for high sensitivity. In the high-pressure range, most internal pores have already been compressed and densified, resulting in minimal deformation and a slow increase in conductive pathways for reduced sensitivity [69, 70]. Besides a rapid response/recovery time of 120/40 ms (Fig. 3d), the rGOA-based pressure sensor can reliably detect subtle pressure of 1 Pa (Fig. 3e) and respond to continuous minute weights (Fig. S24). The rGOA-based pressure sensor can also detect subsequently placed three weights (10, 5, and 1 g) under a high preload from a 200 g weight (or 13.6 kPa), exhibiting high-pressure resolution (Fig. 3f). Meanwhile, the tubelike structure along the Y-direction facilitates the heat dissipation and improves the heat insulation along the other two directions [71], resulting in temperature-insensitive pressure detection in the temperature range from 30 to 50 °C (signal drift < 10%) (Fig. 3g). Although a signal drift of ~ 28% occurs at 130 °C, the sensor maintains excellent cyclic stability (fluctuation < 0.4%) (Fig. S25). Linear fit of the signal drift curves from 30 to 130 °C yields temperature coefficients (TC), defined as *TC* = *(ΔI/I*_*0*_*)/ΔT*, where *ΔT* represents the temperature change. Small TC values of 0.34% °C ^−1^ (30–70 °C) and 0.22% °C ^−1^ (70–130 °C), together with high linearity (*R*^2^ > 0.99), indicate good thermal stability for high-temperature applications (Fig. S26). Although the Joule heating effect during operation causes a gradual increase in the output current of the rGOA (likely due to increased carrier concentration and thermal contraction of the 3D framework for enhanced conductivity [72, 73]), the fluctuation of 0.015 at 0.1 V is far smaller than that caused by pressure (Figs. S27 and S28), indicating negligible impact on sensing performance. To intuitively display the thermal insulation performance of rGOA, it was placed on the Peltier surface (40–120 °C) while rGOA exhibited a significantly lower temperature (30–60 °C) (Fig. S29). The hot-melt adhesive stick placed on the surface of rGOA remains intact at 120 °C while melts on the surface of Peltier due to high temperature (Video S1). Together with stable cycling performance over 5000 or 20,000 loading/unloading cycles under 0.25 or 10 kPa (Figs. 3h and S30), the rGOA-based pressure sensor outperforms most other aerogel-based, MXene-based, and CNT-based flexible piezoresistive pressure sensors (Figs. S31–S32 and Tables S1–S3). The baseline drift during cycling is attributed to gradual stabilization of the rGOA structure and its contact with the interdigitated electrodes. Early microstructural rearrangements improve conductive pathways and electrode contacts, while rapid loading–unloading prevents full recovery, causing a rapid rise in baseline and response. With increasing cycles, the sensing layer stabilizes mechanically and electrically, and the response curve rises more gradually. Conventional fibrous and porous structures in graphene aerogels provide abundant interwoven conductive networks, but their irregular arrangement weakens compressive strength, thereby limiting detection range and stability. In contrast, the anisotropic structure of the rGOA combines high sensitivity with excellent stability. The Nyquist plot of the rGOA from the EIS analysis shows a small semicircle and low total impedance (series resistance *R*_s_ = 31 Ω and polarization resistance *R*_p_ = 94 Ω) in the high-frequency region, indicating efficient charge transfer. After 5000 loading/unloading cycles, the negligible impedance change further confirms the excellent cycling stability of the rGOA-based pressure sensor (Fig. S33).

### Applications in Measuring Human Physiological Activities and Spatial Pressure Detection

**Fig. 4:**
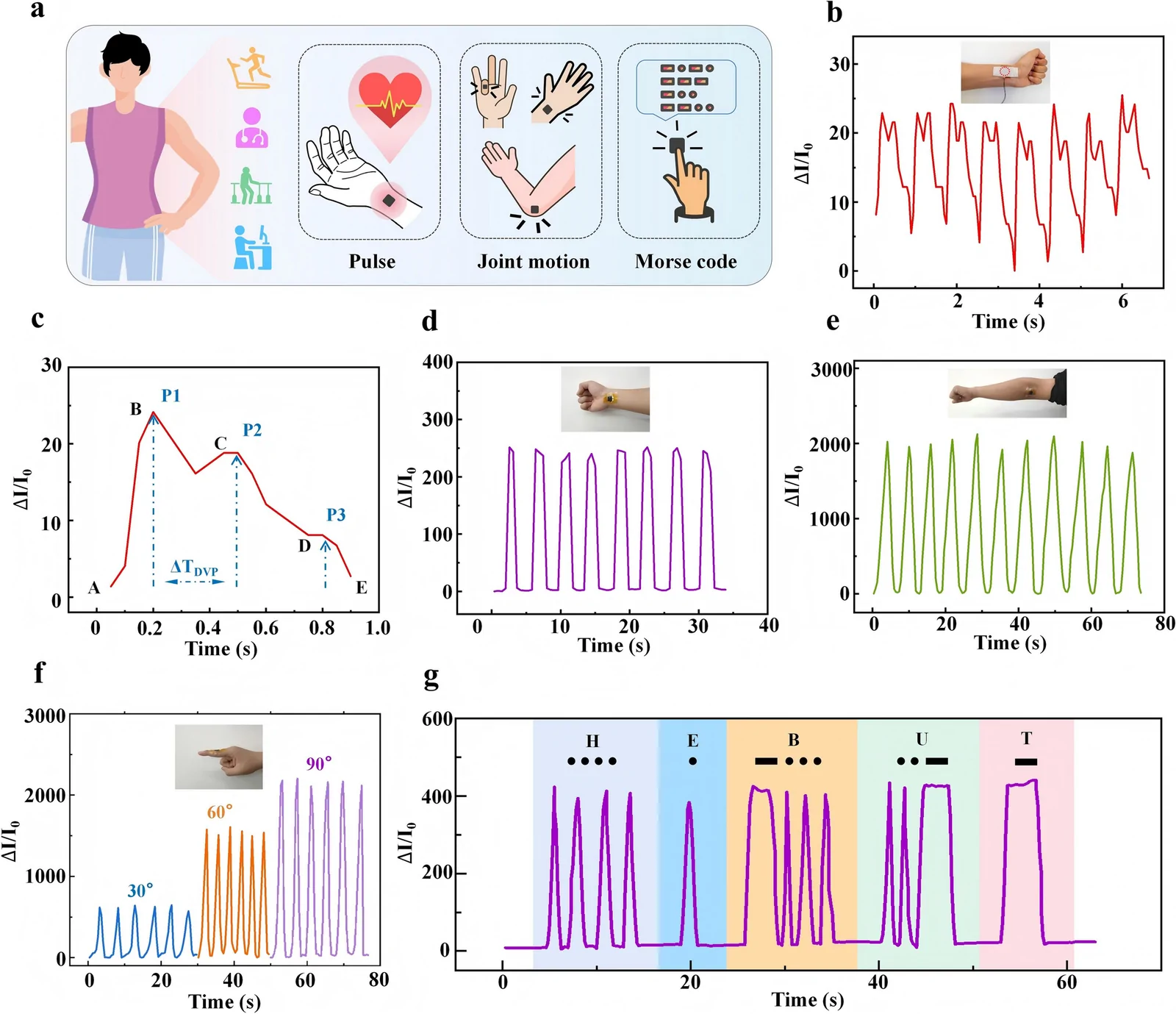
Applications of the rGOA-based pressure sensor to monitor human physiological and motion signals. **a** Schematic to show the applications of physiological/motion monitoring. **b** Cyclic pulse signals measured from the radial artery of the wrist, with **c** a magnified single pulse waveform. The rGOA-based sensor attached to the **d** wrist, **e** elbow, and **f** finger joints to monitor human joint motion. **g** Morse code output of five representative letters, “H,” “E,”, “B,” “U,” and “T,” from finger tapping on the rGOA-based pressure sensor

The flexible rGOA-based pressure sensor with ultra-high sensitivity and rapid response/recovery over a wide pressure range can be utilized to capture different human physiological signals (Fig. 4a). When attached to the wrist of a healthy human subject (25 years old, male), the sensor captures clear characteristic peaks (i.e., percussion P_1_, tidal P_2_, and diastolic P_3_ waves [74]) of pulse signals (Fig. 4b, c), with a heart rate calculated as 67 bpm. The pulse signal remains clear even during motion such as walking and jogging, with the heart rate increased to 87 bpm during jogging (Fig. S34). The long digital volume pulse (Δ*T*_DVP_), defined as the time interval between the P_1_ and P_2_ peaks, reflects the time delay between the forward-traveling pressure wave and the reflected wave in the peripheral arteries, with larger values indicating higher arterial compliance and elasticity [75, 76]. Thus, the measured value of 272 ms indicates healthy arterial properties of the subject. The rGOA-based sensor attached to the wrist, elbow, and finger joints using medical-grade PE tape can also detect clenching/unclenching of the fist (Fig. 4d), bending of the arm (Fig. 4e), and finger bending at 30°, 60°, and 90° (Fig. 4f) for the future rehabilitation of upper-limb functional impairments resulting from stroke or trauma. With a light touch as a dot (·) and a touch and hold for three seconds as a dash (-), the Morse code can be programmed and captured by the rGOA-based sensor, as illustrated by the dynamic signal responses for five representative letters “H,” “E,” “B,” “U,” and “T” (Fig. 4g). The combination of biocompatible PI and PDMS [77] with the rGOA causes no allergic reactions or discomfort even after skin contact for over 24 h, confirming the excellent biocompatibility of the rGOA-based pressure sensor (Fig. S35).

**Fig. 5:**
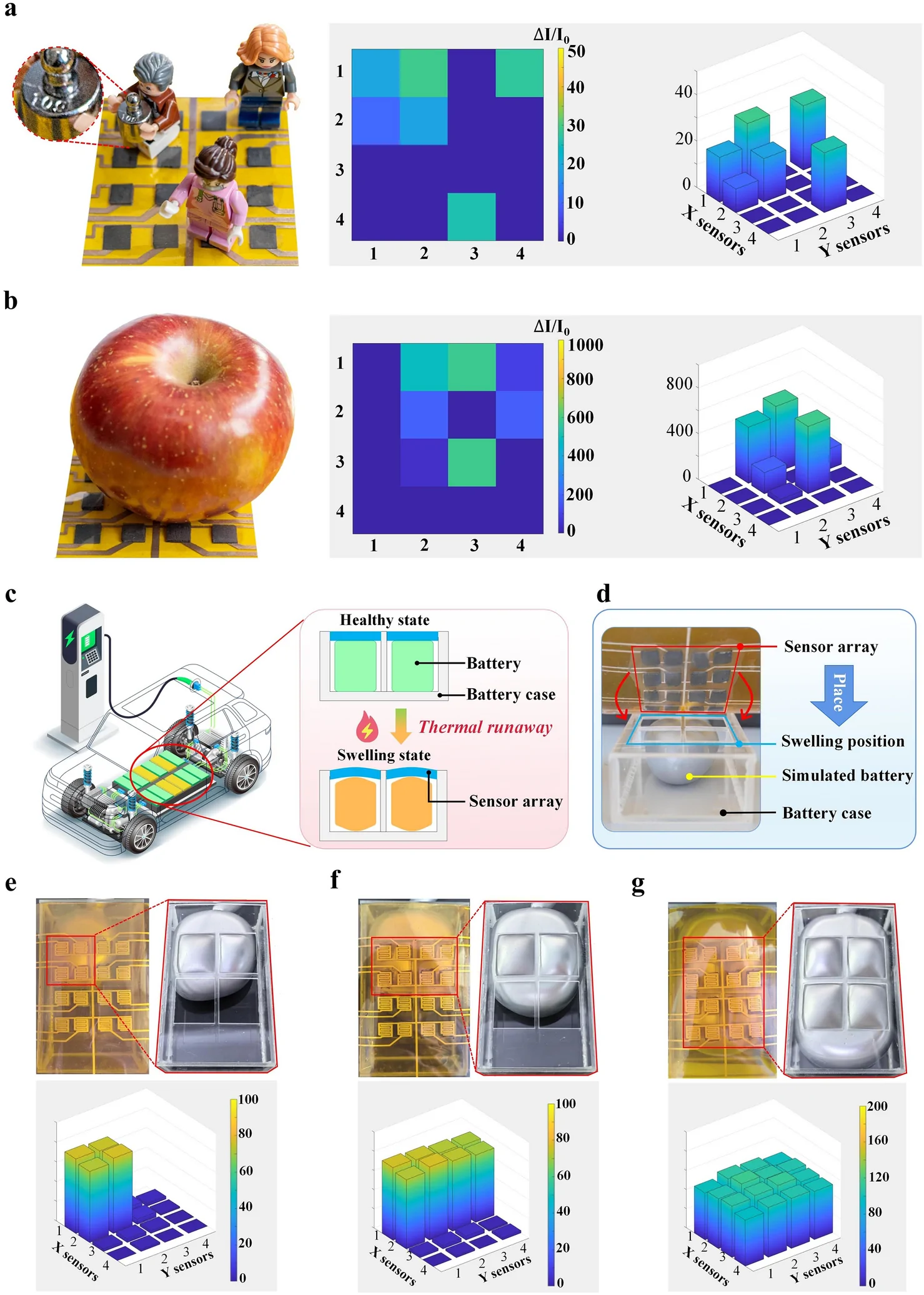
Demonstrations of the rGOA-based pressure sensor array to detect spatial pressure distribution caused by **a** LEGO figurines and **b** an apple, with the corresponding color contrast mapping (middle) and 3D bar graph (right). **c** Schematic diagram and **d** design of the simulated battery swelling experiment for traction batteries in electric vehicles. Swelling of **e** one, **f** two, and **g** four traction batteries (top) and the corresponding pressure distributions (bottom)

Arranging individual rGOA-based pressure sensors into a 4 × 4 array layout can also detect spatial pressure distribution to identify the position and magnitude of the pressure caused by different objects. With the Arduino Mega 2560 to convert the collected 16-channel electrical signals into digital values (Fig. S36), color contrast mapping and 3D bar graph from the MATLAB can further provide direct visualization of the pressure distribution. Besides the identification of varying weights placed on the pressure sensing array (Fig. S37a, b), the pressure distribution caused by a toy car (with four wheels in contact) (Fig. S37c), an irregularly shaped key (Fig. S37d), three LEGO minifigures (Fig. 5a), and the apple (Fig. 5b) is also captured (Video S2). The 4 × 4 rGOA-based pressure sensing array (Fig. S38) with exceptional thermal stability can also allow real-time monitoring of battery volume in traction battery packs inside electric vehicles during charging, discharging, and normal use. It can provide timely warnings when irreversible battery swelling occurs at elevated temperatures (> 45 °C) due to electrolyte decomposition and gas generation [78, 79], thereby preventing thermal runaway accidents (Fig. 5c). With an inflating balloon inside an acrylic battery case (Fig. S39) to simulate battery swelling (Fig. 5d), the simulated swelling from one, two, and four battery cells is captured and visualized as a 3D bar graph (Fig. 5e–g). With a battery volume expansion of approximately 2% (the initial stage of swelling) defined as the warning threshold [80], an alarm function is implemented in the MATLAB-based pressure monitoring interface (Fig. S40). Future work will establish an experimental platform for direct testing on lithium-ion batteries.

### Applications of the Smart Manipulator for Teleoperation with Force Feedback

**Fig. 6:**
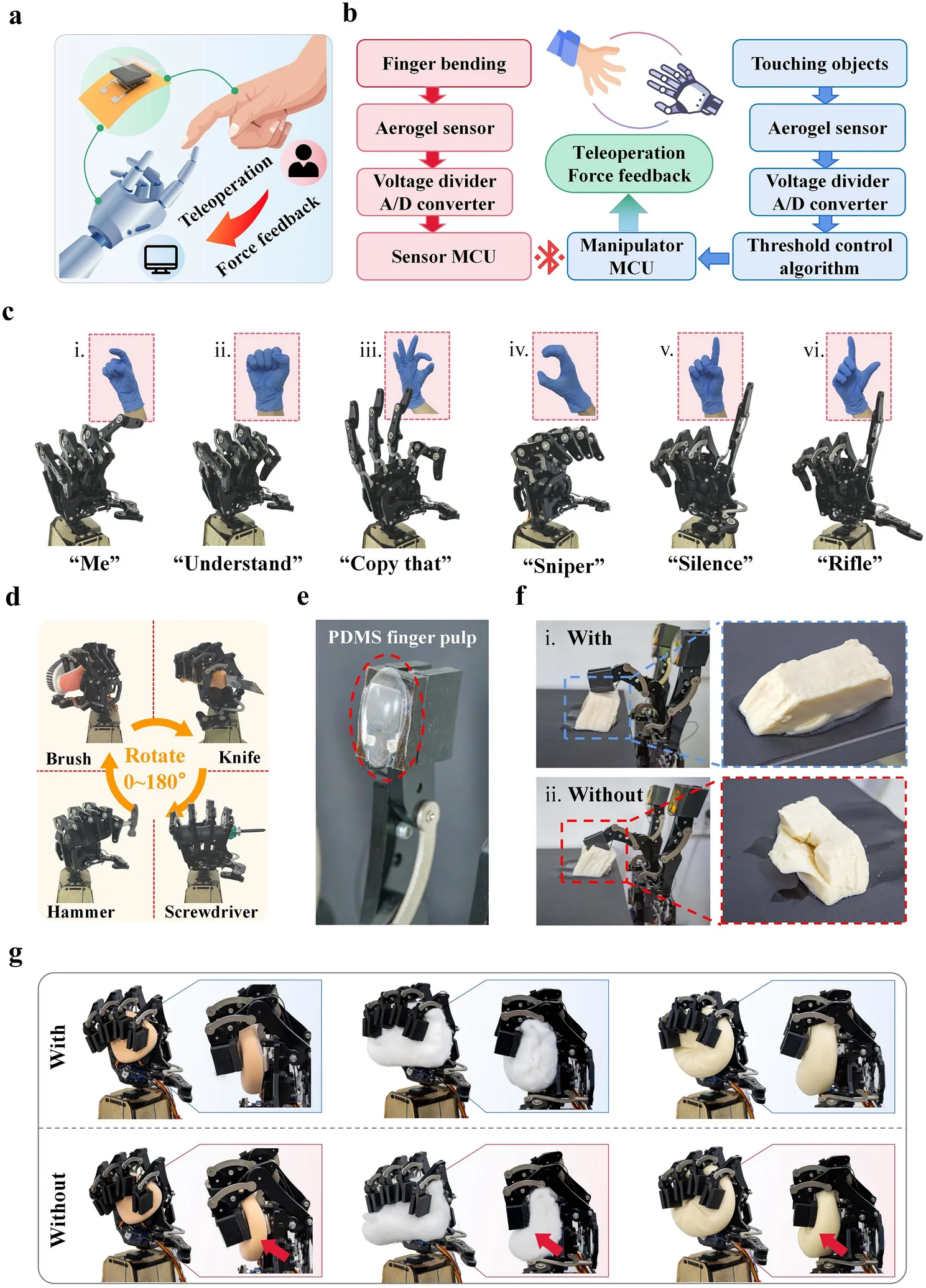
Demonstration of rGOA-based pressure sensors in a manipulator teleoperation system with force feedback. **a** Schematic illustrations and **b** the principal block diagram of the manipulator teleoperation system. **c** Real-time control of the manipulator through teleoperation to show gestures of “Me”, “Understand”, “Copy that”, “Sniper”, “Silence”, and “Rifle.” **d** Real-time control of the manipulator through teleoperation to grasp brushes, knives, screwdrivers, hammers, and rotate them from 0° to 180°. **e** rGOA-based sensor with fingertip-pulp-shaped PDMS on the surface of the manipulator finger. **f** A comparison of the manipulator (i) with and (ii) without feedback modules to touch tofu and **g** grasp powder puff, cotton, and steamed bun

The bending motion obtained from the rGOA-based sensors attached to the joints of the finger can be wirelessly transmitted to the manipulator for control and manipulation, whereas the pressure sensors on the fingers of the manipulator can provide force feedback (Figs. 6a, b and S41). Besides detecting simple hand gestures (e.g., “5” ,“4”, “3” ,“2” ,“1”, and “0”) (Fig. S42), tactical sign language essential for the communication between soldiers in the battlefield is also demonstrated for the representative gestures: “Me”, “Understand”, “Copy that”, “Sniper”, “Silence”, and “Rifle” (Fig. 6c and Video S3).

With an additional rGOA-based sensor on the nail of the middle finger for controlling the rotary servo, the manipulator can rotate in the range from 0° to 180° while firmly gripping brushes, knives, screwdrivers, and hammers, collaborating with human to complete brushing and cutting in the workshop (Fig. 6d and Video S4). The five rGOA-based pressure sensors on the fingertips of the manipulator monitor the pressure in real time during grasping and compare it with the preset value to ensure stable grasping, while preventing the object from receiving too much pressure. Further integrating a fingertip-pulp-shaped PDMS on the sensor surface enhances multi-directional force detection (Figs. 6e and S43) and minimizes gaps between the sensor and target objects (Fig. S44) [81–83]. Touching the soft tofu with the manipulator with and without the rGOA-based sensing module directly demonstrates the importance of the force-feedback system provided by the sensor to stabilize grasping (Video S5). When the measured signal from the rGOA-based pressure sensor on the manipulator fingertip exceeds the preset safety threshold, the servo stops to prevent deforming and damaging the tofu (Fig. 6f). Similar results are observed in manipulating the other fragile daily objects (e.g., powder puff, cotton, and steamed bun) (Fig. 6g). These systems can also be adapted for other human*–*machine interaction applications such as ocean exploration and industrial manipulation.

### Application of the Smart Manipulator for Object Recognition in the Kitchen

**Fig. 7:**
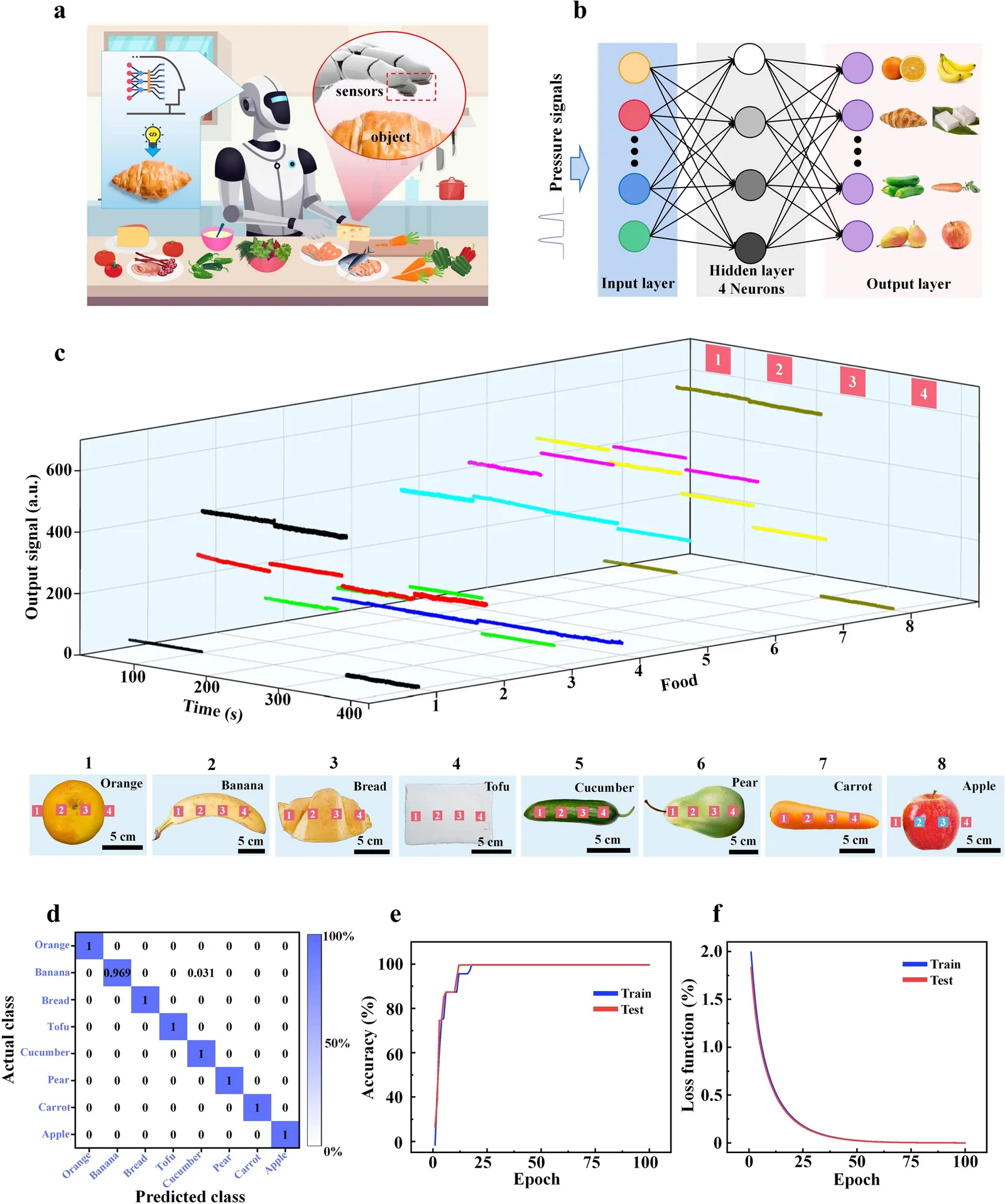
Application of the rGOA-based object recognition system in the kitchen. Schematic showing **a** rGOA-based kitchen robot and **b** BP neural network for food recognition, with the corresponding signal outputs from grasping **c** orange, banana, bread, tofu, cucumber, pear, carrot, and apple (1, 2, 3, and 4 representing the contact sensor on the index, middle, ring, and pinky fingers). **d** Confusion matrix for kitchen object recognition. **e** Training/testing accuracy and **f** loss function curves as a function of the epoch

Taken together with advanced machine learning algorithms, the manipulator with the rGOA-based pressure sensor (rGOA manipulator) can autonomously distinguish food ingredients based on tactile recognition to provide personalized services for humans in the kitchen (Fig. 7a). The pressure responses of the rGOA manipulator to eight food ingredients (i.e., orange, banana, bread, tofu, cucumber, carrot, pear, and apple; with four sampling positions from four fingers for each) serve as the input signals for the BP neural network (Fig. 7b), with 4 nodes in the hidden layer as in the literature [84]. With sensors on the index, middle, ring, and pinky fingers of the rGOA manipulator to capture the pressure signals (Fig. 7c), 1000 sets of data for each of the eight foods have been collected to differentiate hardness and shape, with 70% for training and 30% for validation (learning rate of 0.01 and 100 epochs). The manipulator’s fingers are programmed to rotate 90° to touch the food placed on a fixed planar platform. Due to variations in the surface contour of the food, the fingers’ servos experience different levels of obstruction. The varying hardnesses of the food also lead to distinct responses from the rGOA-based sensors on the four fingers, providing the neural network model with high accuracy and fast convergence. As demonstrated in the confusion matrix, the recognition accuracy for the eight foods reaches 99.58% (Fig. 7d). The accuracy from both the training and testing datasets continuously increases to 99.58% after only 19 epochs (Fig. 7e), while the loss decreases continuously and approaches 0 at 100 epochs (Fig. 7f). The feature-space visualization, obtained by projecting high-dimensional features onto two principal components using principal component analysis, clearly shows that the eight trained food categories form distinct, non-overlapping clusters, indicating strong intrinsic separability of the collected pressure signals (Fig. S45a). The new unseen food samples of peeled banana lie close to the banana cluster in the feature space and are therefore classified as bananas by the model (Fig. S45b). This result demonstrates that the model generalizes the underlying feature distribution, eliminating the concerns of parameter overfitting. The rGOA manipulator exhibits excellent sensitivity, a wide detection range, and temperature/humidity stability, minimizing performance fluctuations in the presence of smoke, steam, moisture, or temperature changes in the kitchen for future smart kitchen robots.

## Conclusions

In summary, this work has reported the design, characterization, and demonstration of a high-performance flexible pressure sensor based on the rGO aerogel with a highly porous 3D structure. The rGOA-based pressure sensor shows an ultra-high sensitivity of 698.960 kPa^−1^, a low limit (1 Pa) and broad range (1 Pa to 100 kPa) of detection, fast response/recovery (120/40 ms), and outstanding stability over 20,000 loading/unloading cycles. Besides monitoring wrist pulse and joint motions (e.g., wrist, finger, and elbow), the flexible rGOA-based pressure sensors configured into an array layout as a smart artificial e-skin can also detect spatial pressure distribution for human–machine interactions, traction battery health, and intelligent prostheses. Furthermore, integrating rGOA-based sensors with signal processing modules and a manipulator in a remote operation system allows for gesture recognition. Taken together with a machine learning algorithm based on the BP neural network, the rGOA manipulator can be used for object recognition in the kitchen, providing opportunities for multimodal recognition and smart home robots. More importantly, the rGOA-based pressure sensor offers a low-cost and high-performance sensing solution. By leveraging its flexibility and customizable array design, it can be readily integrated with wearable devices and commercial robots with a high degree of adaptability. It is possible to further reduce the sensor’s size and weight, miniaturize the hardware circuits integration, and enhance biocompatibility and stability under complex environments before the deployment of rGOA sensors in real-world applications.

## Supplementary Information

Below is the link to the electronic supplementary material.

Supplementary file1 (MP4 5155 KB)

Supplementary file2 (MP4 744 KB)

Supplementary file3 (MP4 5513 KB)

Supplementary file4 (MP4 6888 KB)

Supplementary file5 (MP4 4388 KB)

Supplementary file6 (DOCX 17218 KB)
