# Supplementary material for: Graphene Aerogel-Based Flexible Pressure Sensor for Physiological Signal Detection and Human–Machine Interaction
Source: Nanomicro Lett. 2026 Mar 27;18:308. doi: 10.1007/s40820-026-02109-8 (PMC13031469; doi:10.1007/s40820-026-02109-8)
Supplement: Supplementary file 6 — Supplementary file6 (DOCX 17218 KB) [file 40820_2026_2109_MOESM6_ESM.docx]

Supporting Information for

**Graphene Aerogel-Based Flexible Pressure Sensor for Physiological Signal Detection and Human-Machine Interaction**

Zihan Wang^1^, Zeshang Zhao^1^, Qiyang Tu^1^, Chengpeng Yao^2^, Zhao Liu^2^, Chengzhi Zhou^1^, Luxiang Xu^1^, Shijie Guo^1^, Chuizhou Meng^3,^ *, Gaofeng Shao^4,^ *, Huanyu Cheng^5,^ *, Li Yang^2,^ *

^1^ State Key Laboratory for Reliability and Intelligence of Electrical Equipment, Hebei Key Laboratory of Smart Sensing and Human-Robot Interaction, School of Mechanical Engineering, Hebei University of Technology, Tianjin 300401, P. R. China

^2^ State Key Laboratory of Reliability and Intelligence of Electrical Equipment, School of Health Sciences and Biomedical Engineering, Hebei University of Technology, Tianjin 300130, P. R. China

^3^ Research Institute of Wearable Electronic Materials and Devices, School of Materials Science & Engineering, Zhejiang Sci-Tech University, Hangzhou 310018, P. R. China

^4^ School of Chemistry and Materials Science, Nanjing University of Information Science & Technology, Nanjing, 210044, P. R. China

^5^ Department of Engineering Science and Mechanics, The Pennsylvania State University, University Park, 16802, USA

*Corresponding authors. E-mail: [2018108@hebut.edu.cn](mailto:2018108@hebut.edu.cn) (Chuizhou Meng); [gfshao@nuist.edu.cn](mailto:gfshao@nuist.edu.cn) (Gaofeng Shao); [huanyu.cheng@psu.edu](mailto:huanyu.cheng@psu.edu) (Huanyu Cheng); [yangli5781@126.com](mailto:yangli5781@126.com) (Li Yang)

**Supplementary Figures and Tables**

**
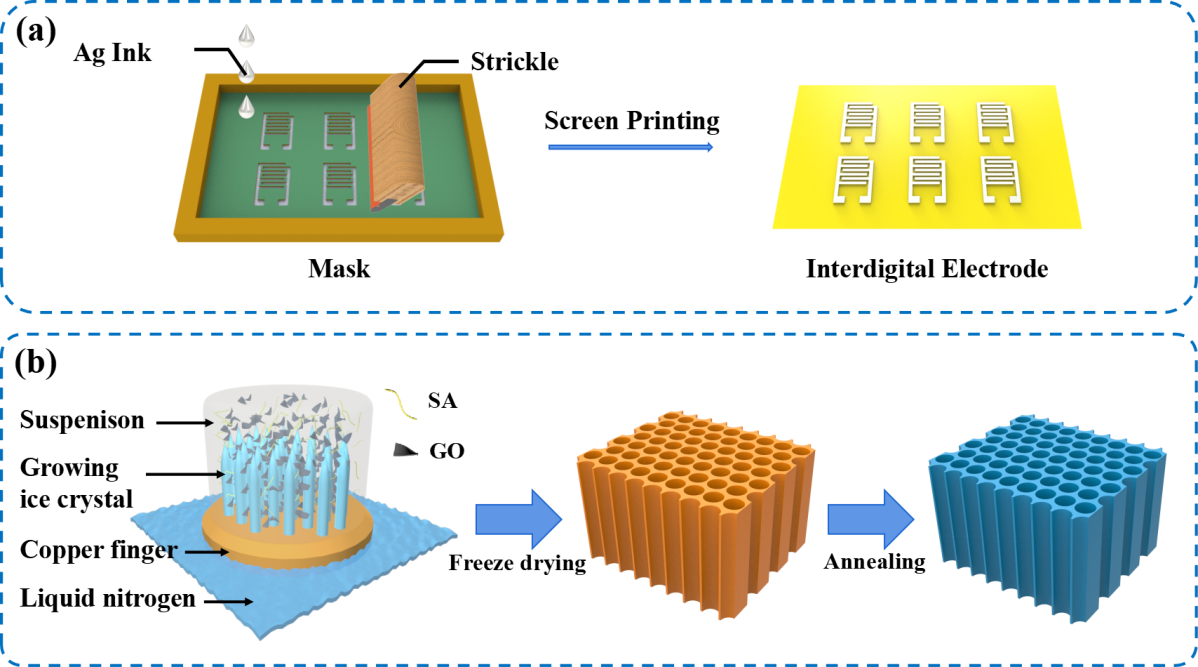
**

**Fig. S1** Schematic diagram of the preparation process of (**a**) Ag interdigital electrode and (**b**) rGO aerogel

**
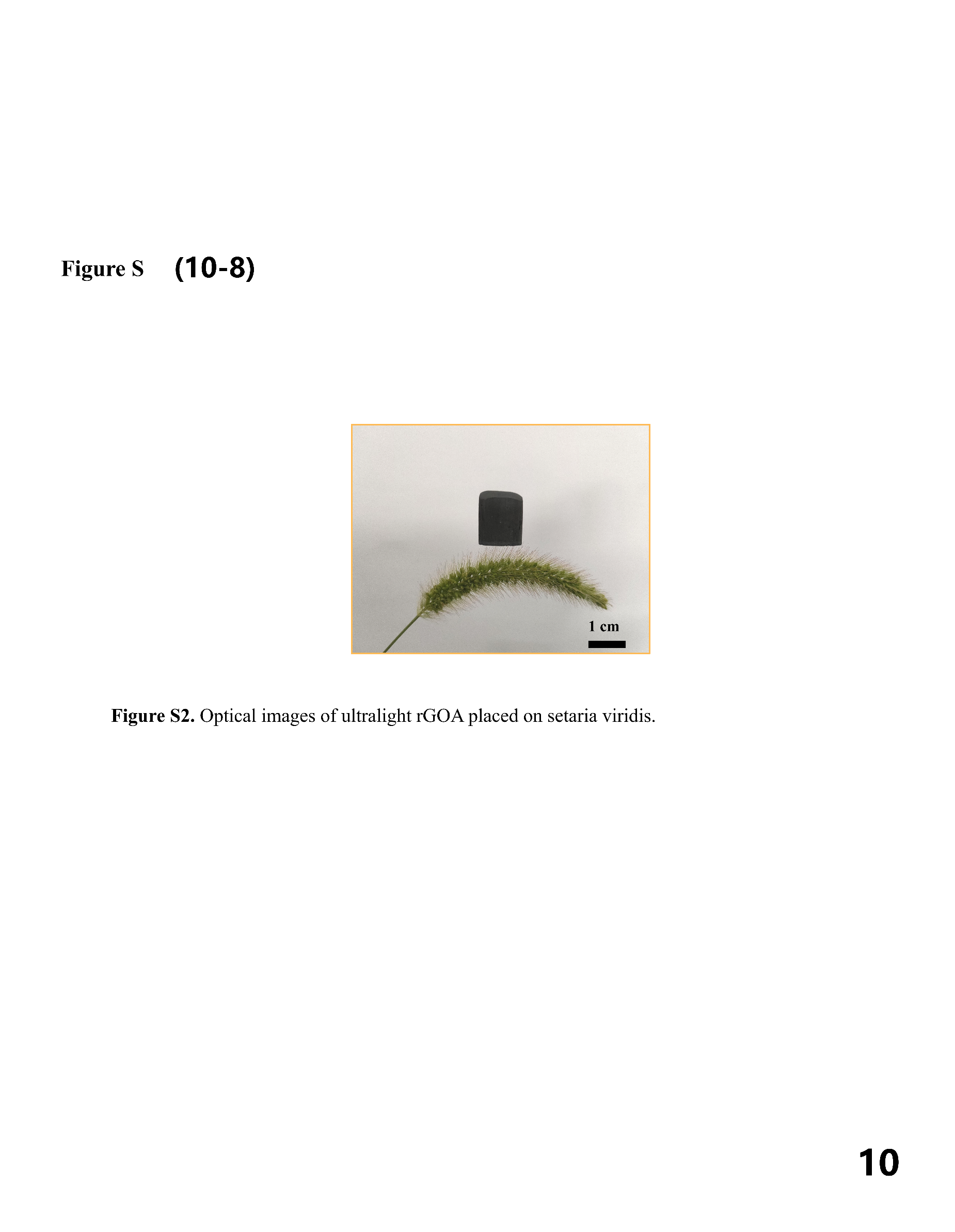
**

**Fig. S2** Optical images of ultralight rGOA placed on setaria viridis


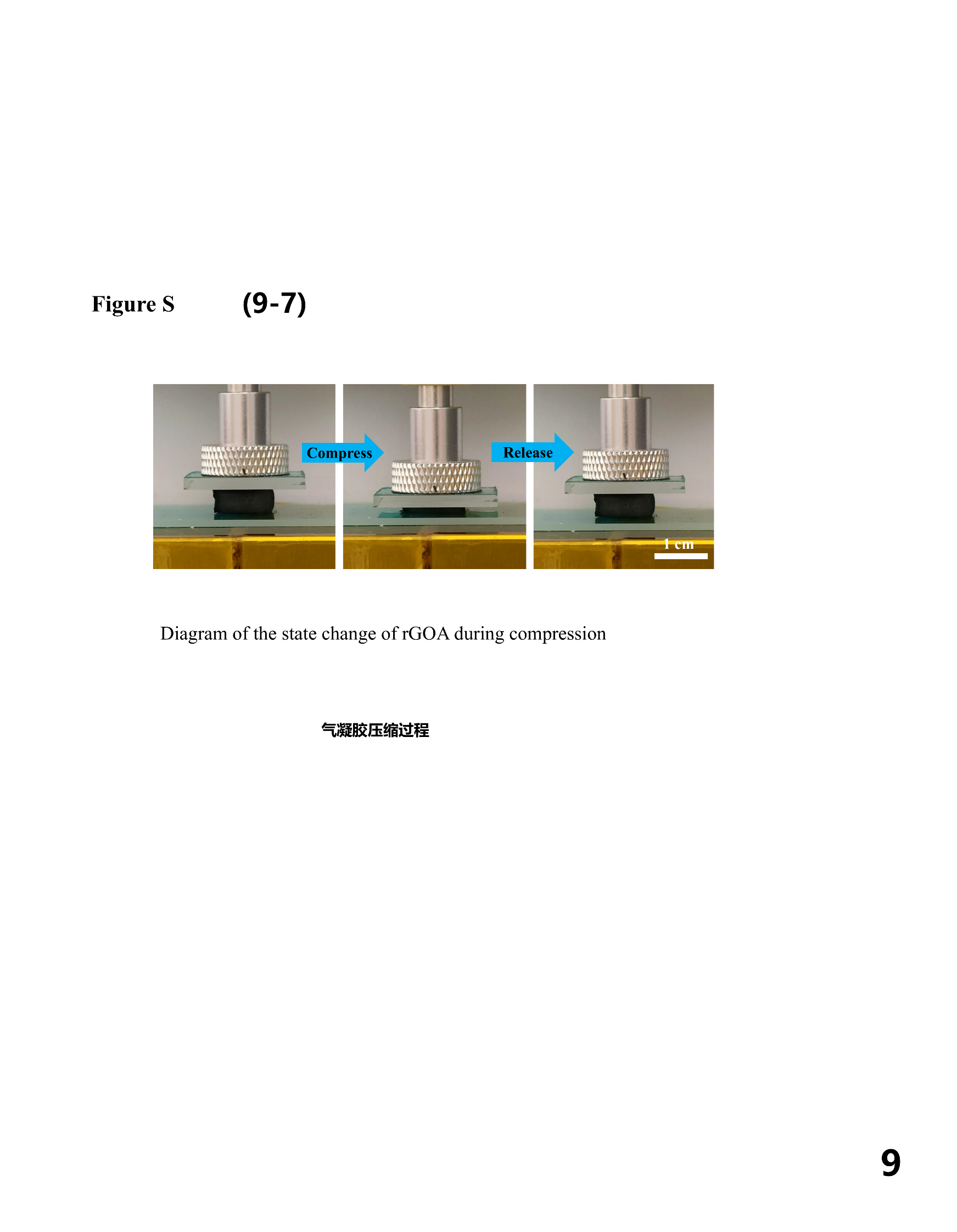


**Fig. S3** Photographs of rGOA during compression and after release


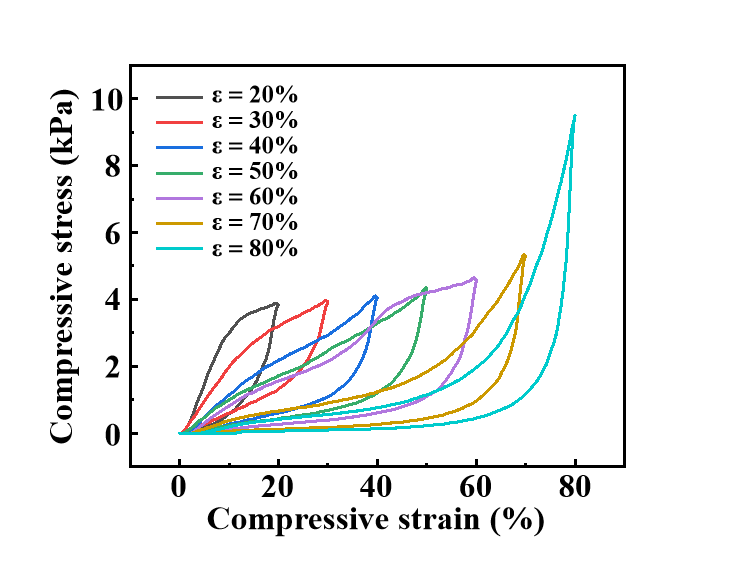


**Fig. S4** Compressive stress-strain curves of the rGOA under the applied strain from 20% to 80% along the Y-direction


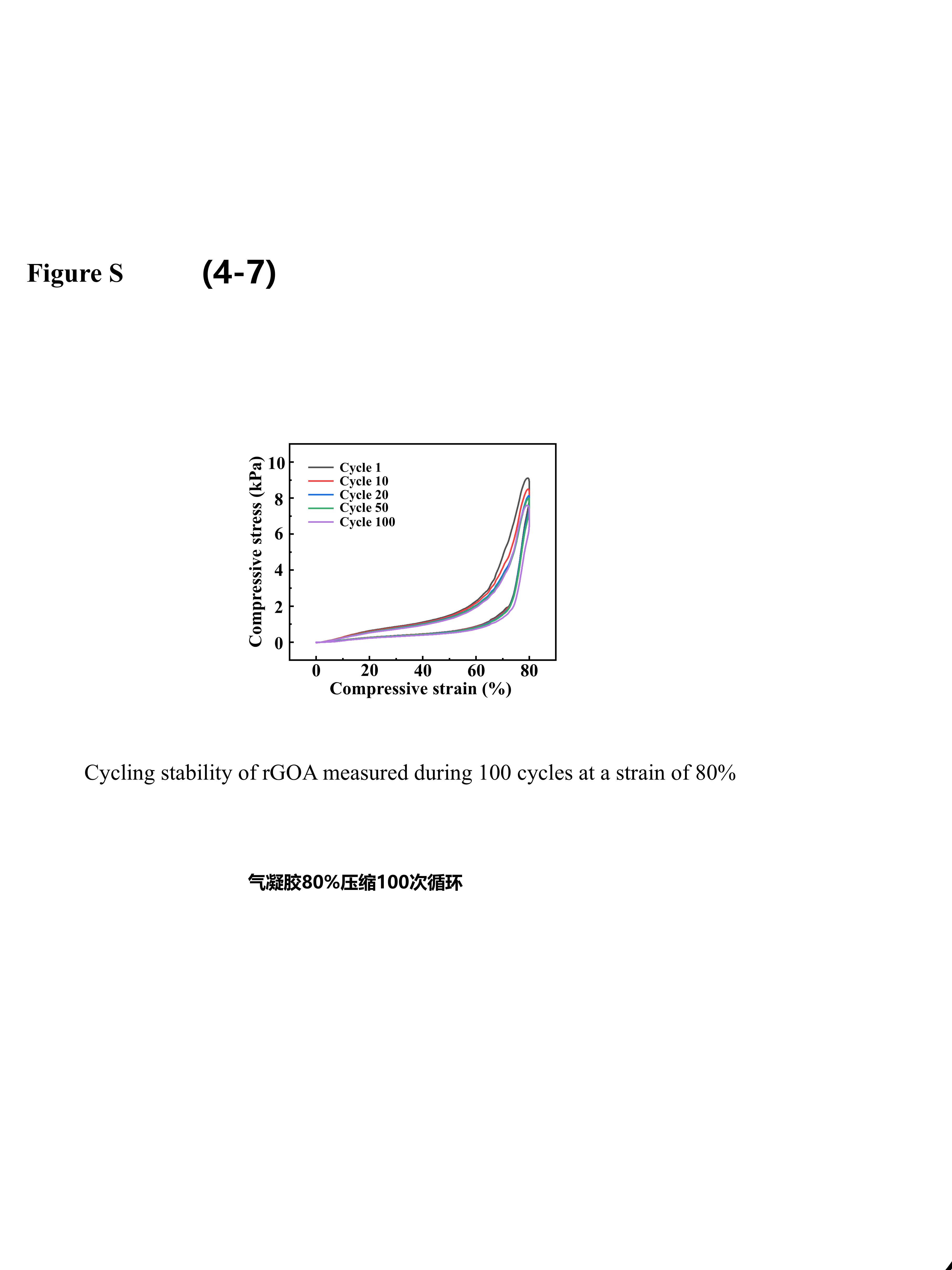


**Fig. S5** Cycling stability of rGOA measured during 100 cycles for a compressive strain of up to 80%


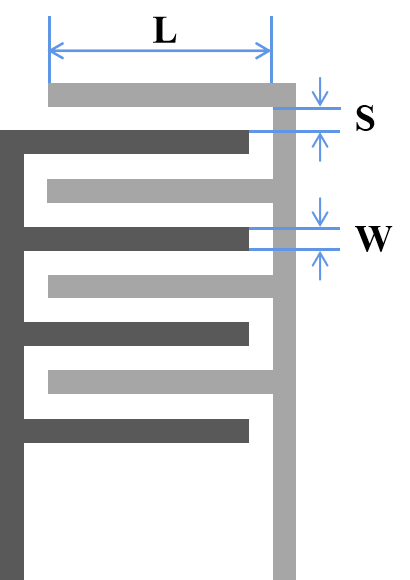


**Fig. S6** Rectangular interdigital electrode structure, with finger length (L), finger spacing (S), and finger width (W) labeled


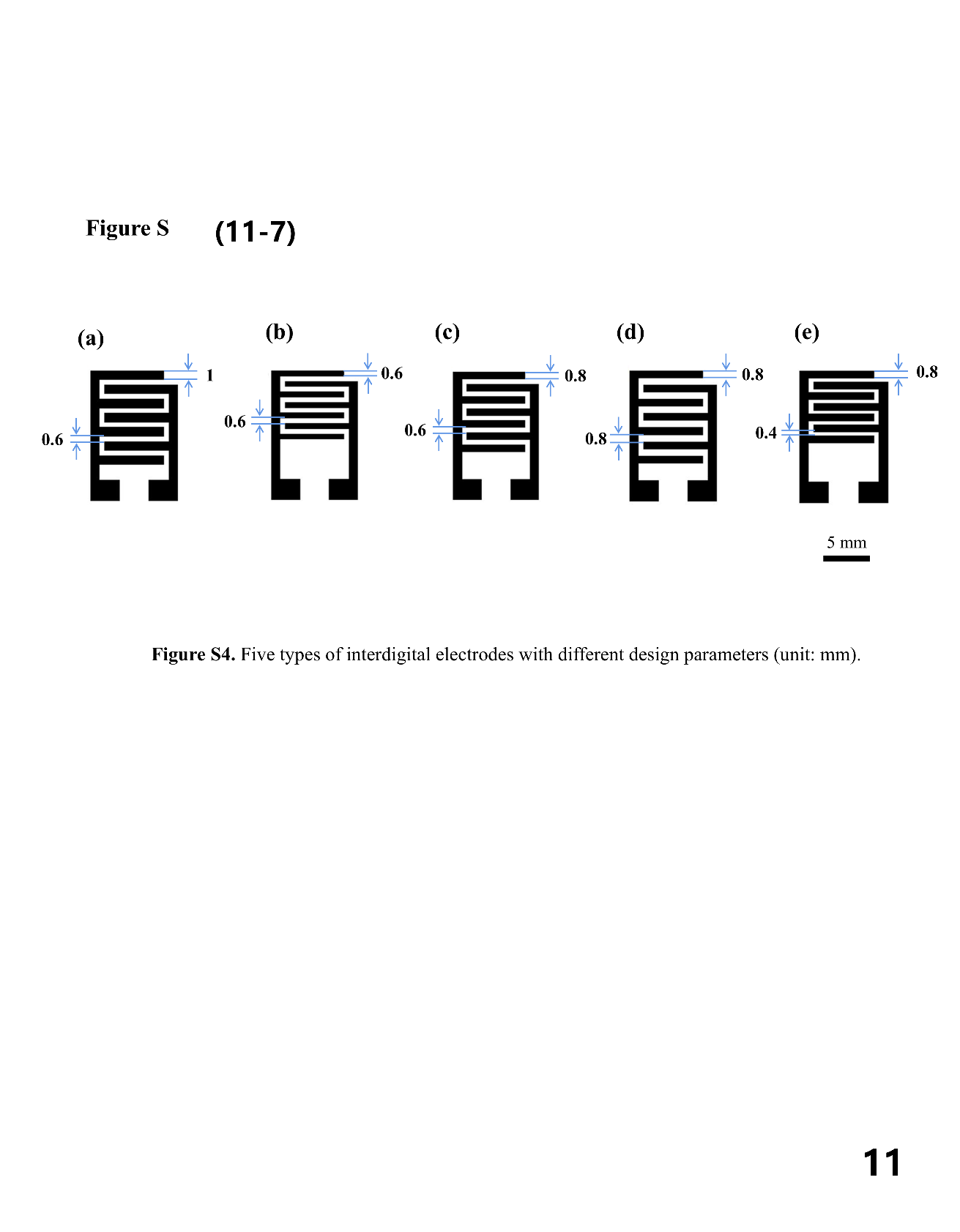


**Fig. S7** Schematic showing the interdigital electrode with different design parameters


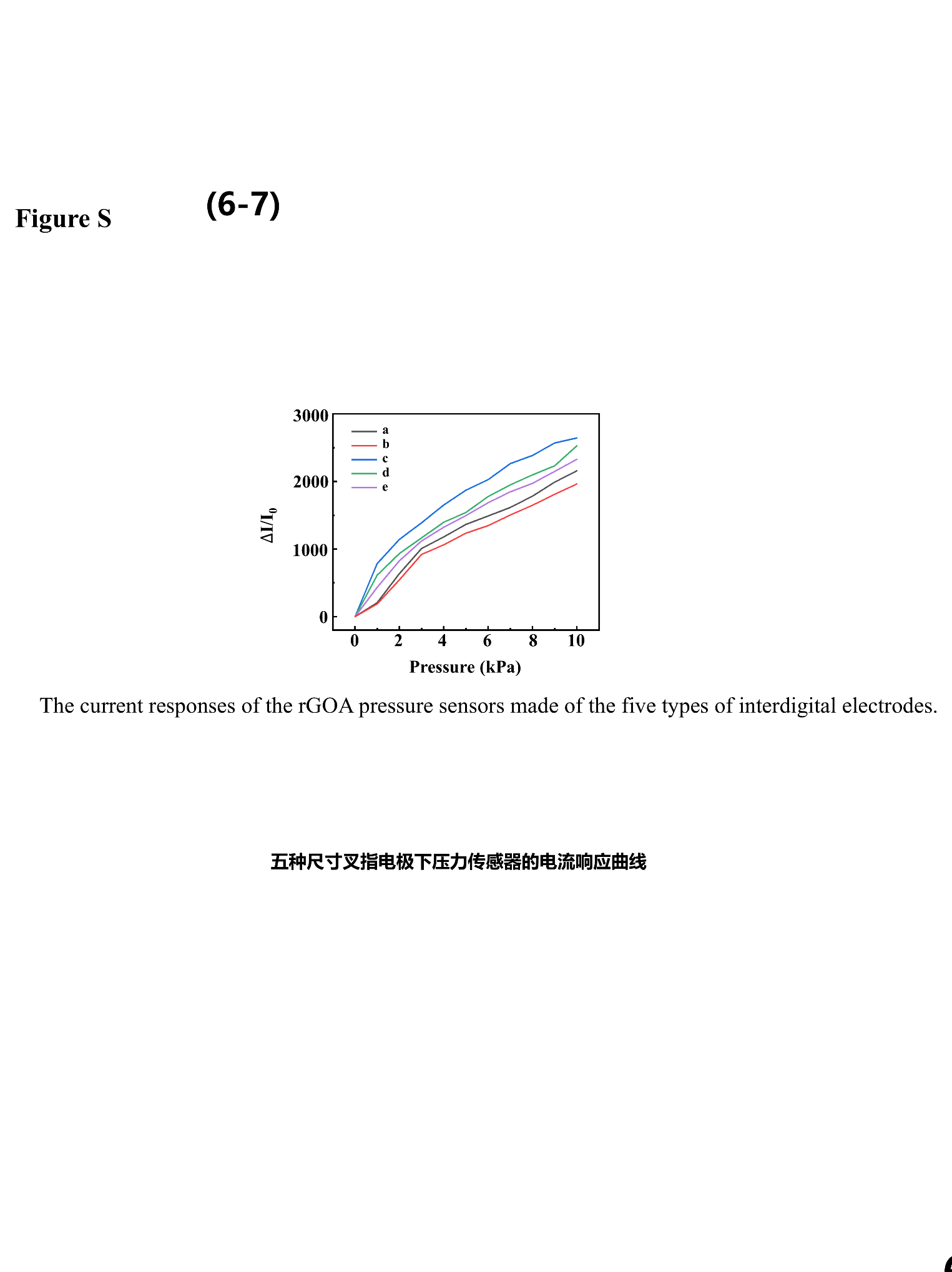


**Fig. S8** Comparison in the current response between the rGOA-based pressure sensors with five different interdigital electrodes


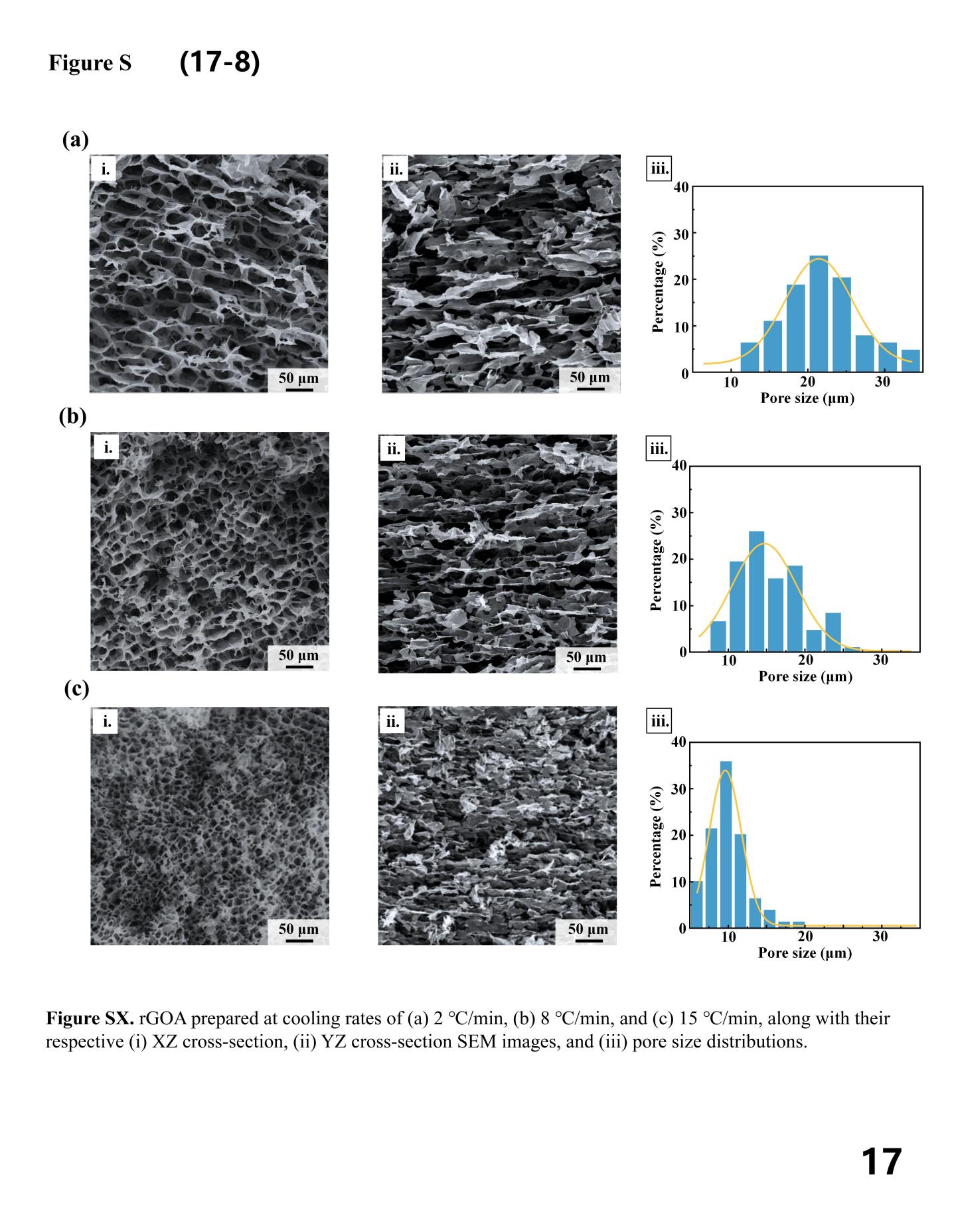


**Fig. S9** rGOA prepared at cooling rates of (**a**) 2 ℃/min, (**b**) 8 ℃/min, and (**c**) 15 ℃/min, along with their respective (i) XZ cross-section, (ii) YZ cross-section SEM images, and (iii) pore size distributions


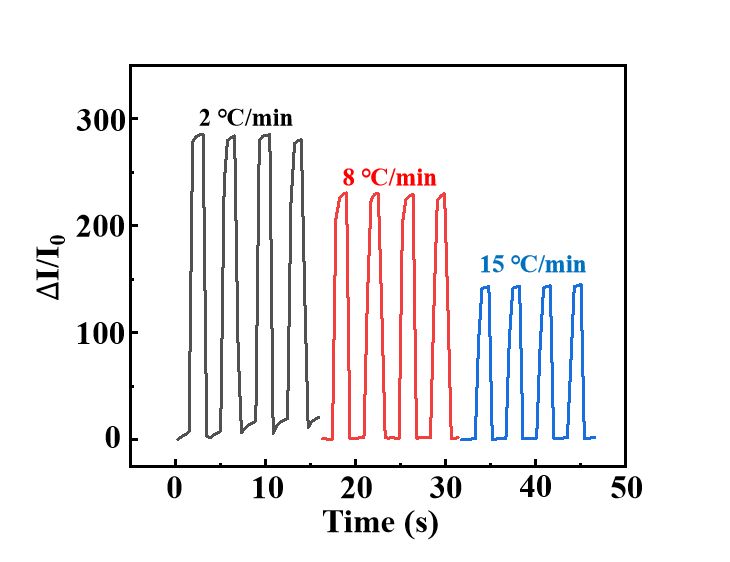


**Fig. S10** Current responses of rGOA-based pressure sensors prepared at different cooling rates (i.e., 2, 8, and 15℃/min) under SA:GO=2:3 and a pressure load of 1 kPa


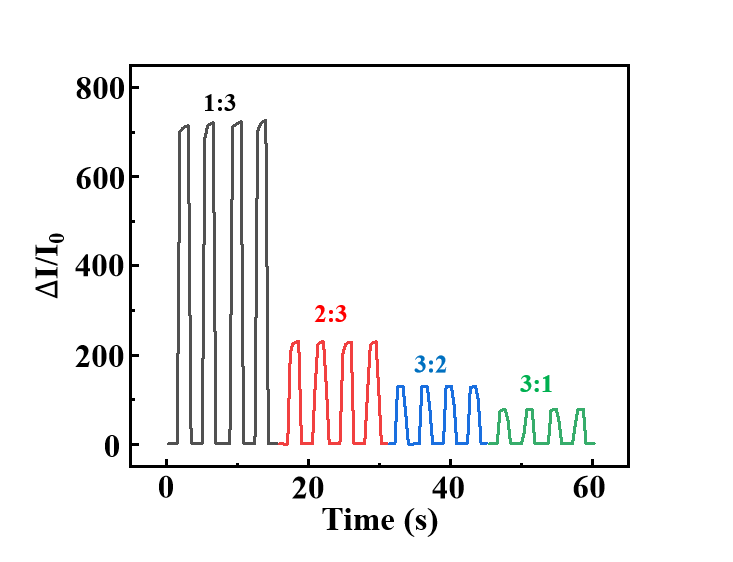


**Fig. S11** Current responses of rGOA-based pressure sensors with different SA:GO ratios (1:3, 2:3, 3:2, 3:1) under a pressure load of 1 kPa and a cooling rate of 8℃/mi


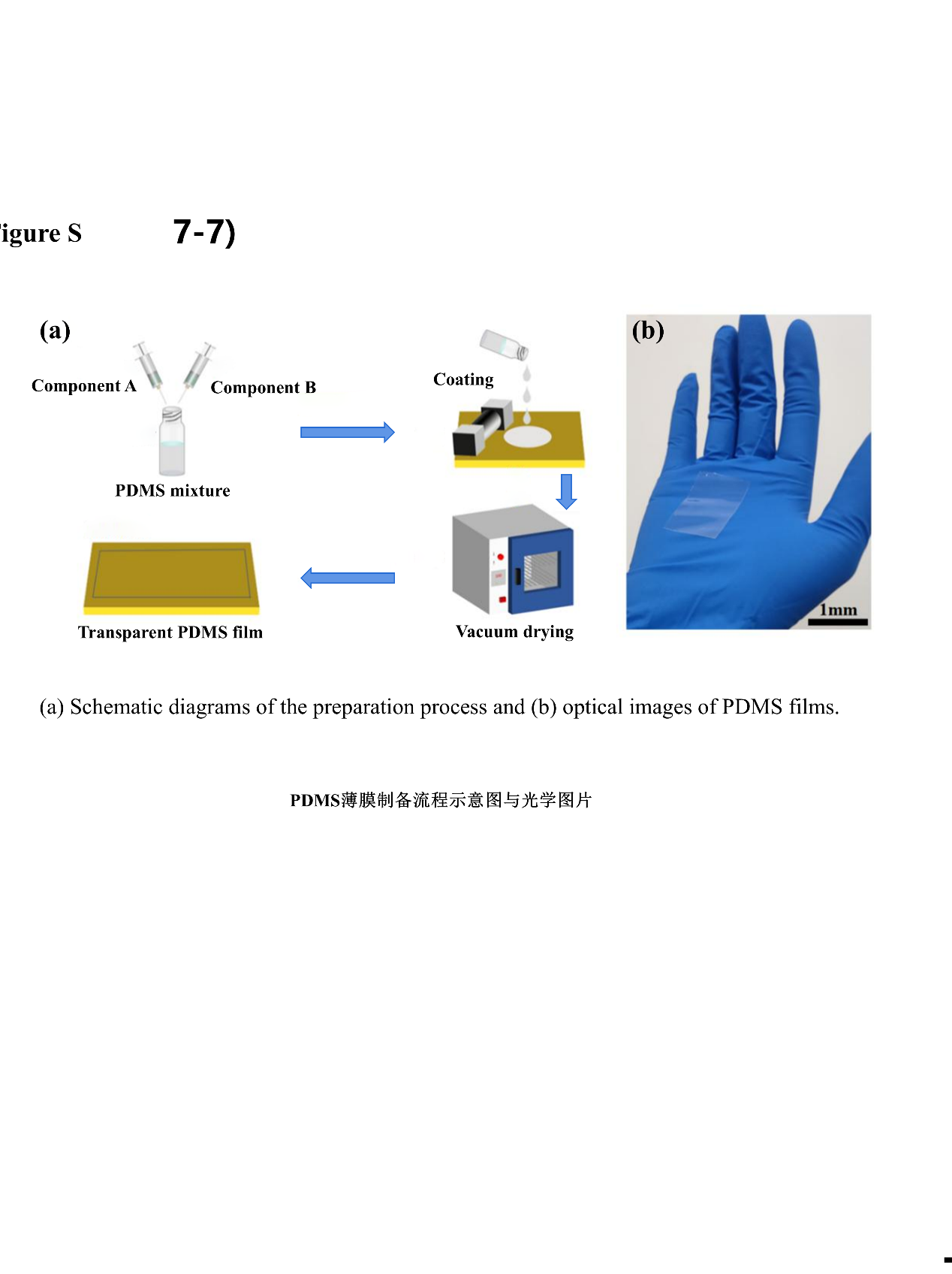


**Fig. S12** (**a**) The preparation process and (**b**) optical image of the transparent PDMS film


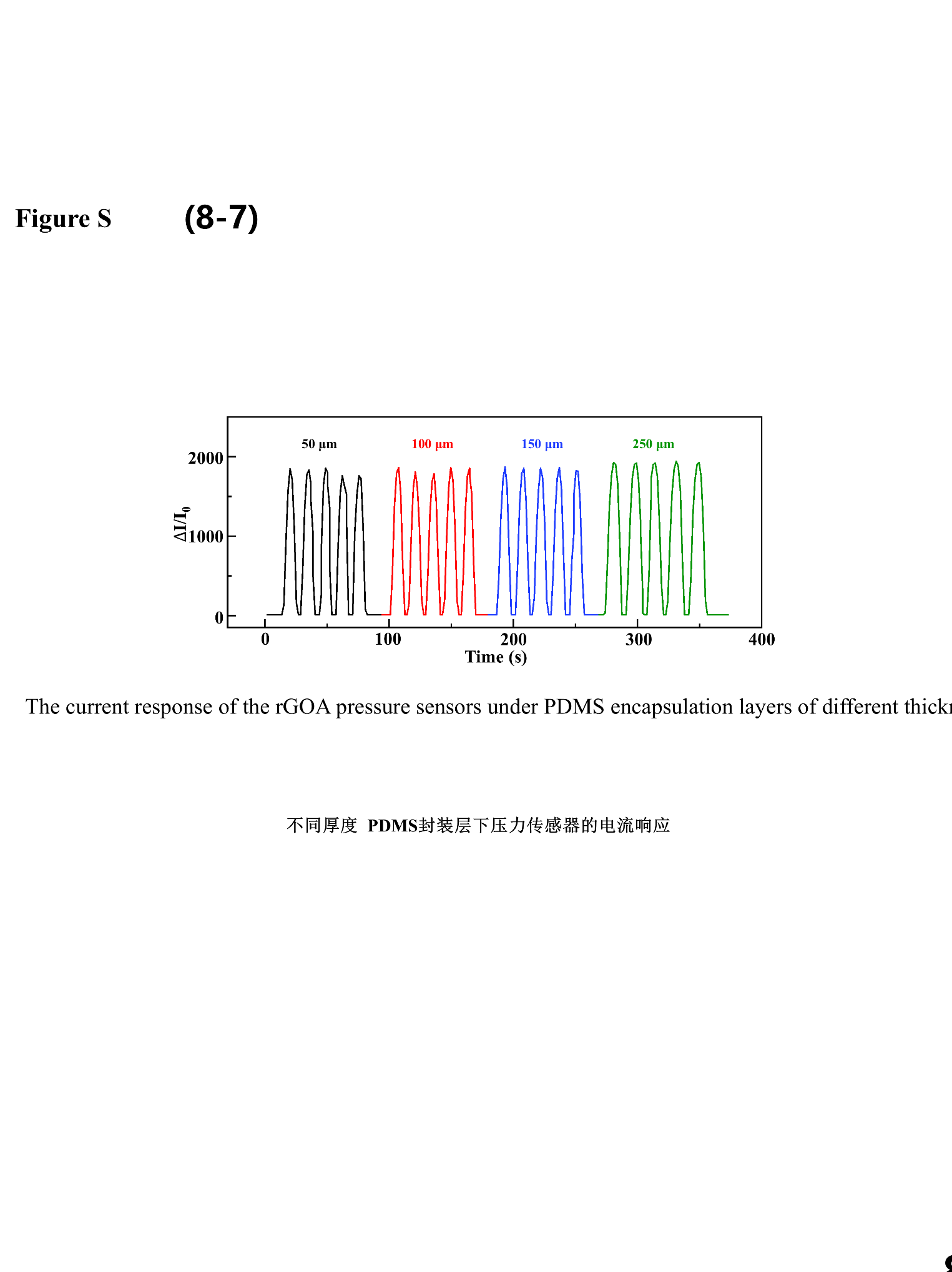


**Fig. S13** Comparison in the current response of the rGOA-based pressure sensors encapsulated by PDMS with different thicknesses


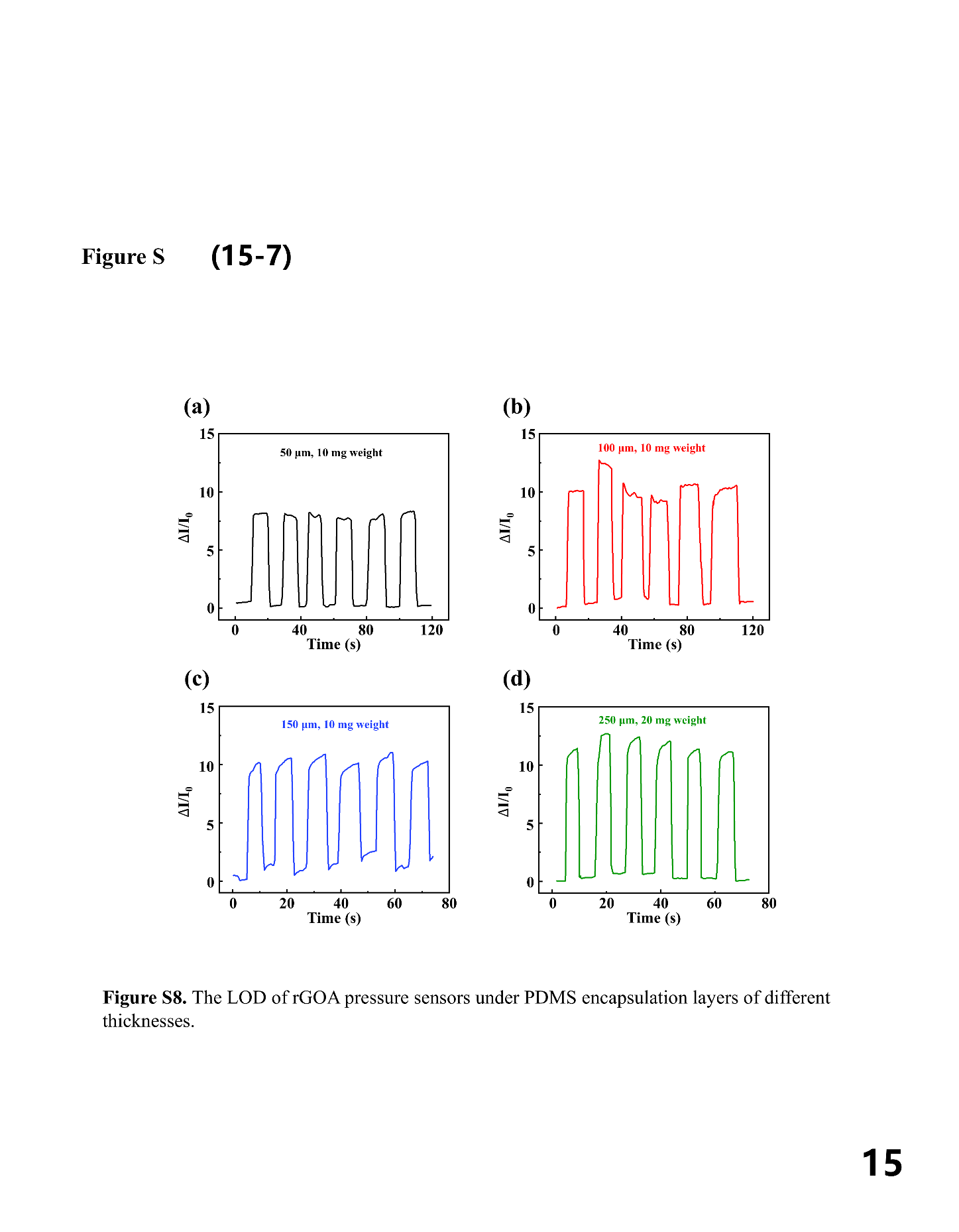


**Fig. S14** Demonstration of the pressure sensing near the LOD for the rGOA-based pressure sensors encapsulated by PDMS with different thicknesses: (**a**) 50, (**b**) 100, (**c**) 150, and (**d**) 200 μm


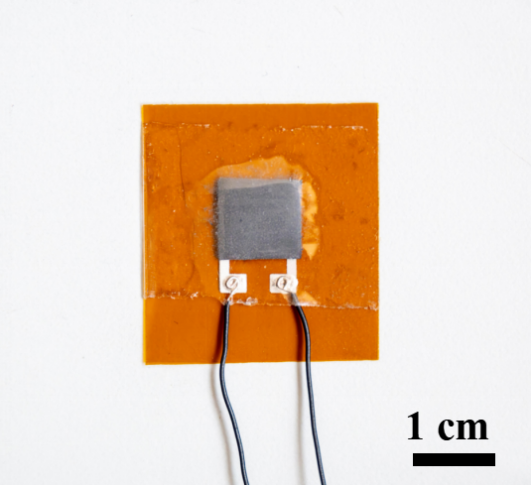


**Fig. S15** Photograph of the rGOA-based pressure sensor


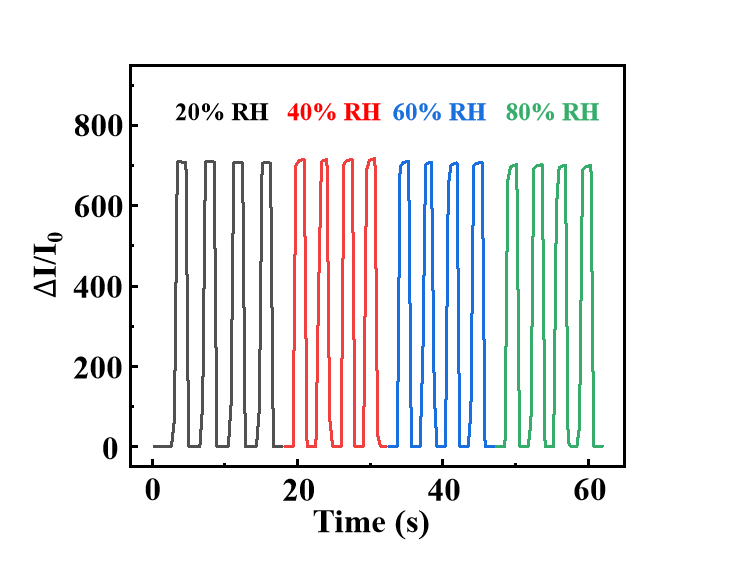


**Fig. S16** The current response of the rGOA-based pressure sensor in the RH ranging from 20% to 80%


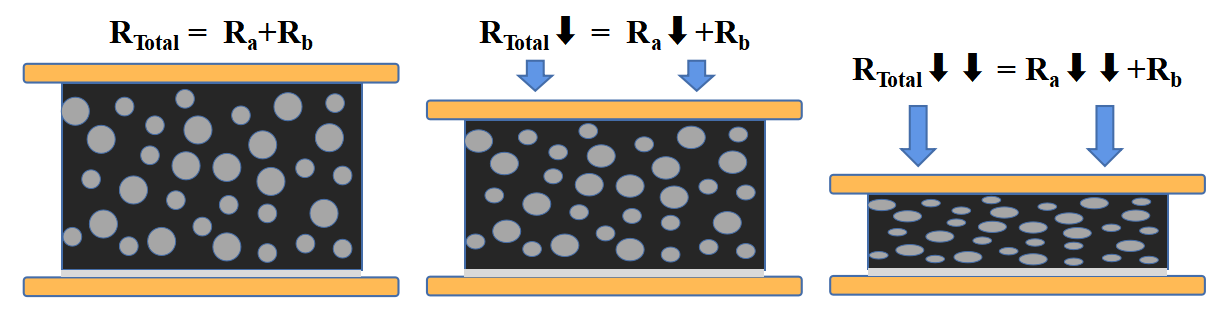


**Fig. S17** Schematic diagram of the working principle of the rGOA-based pressure sensor


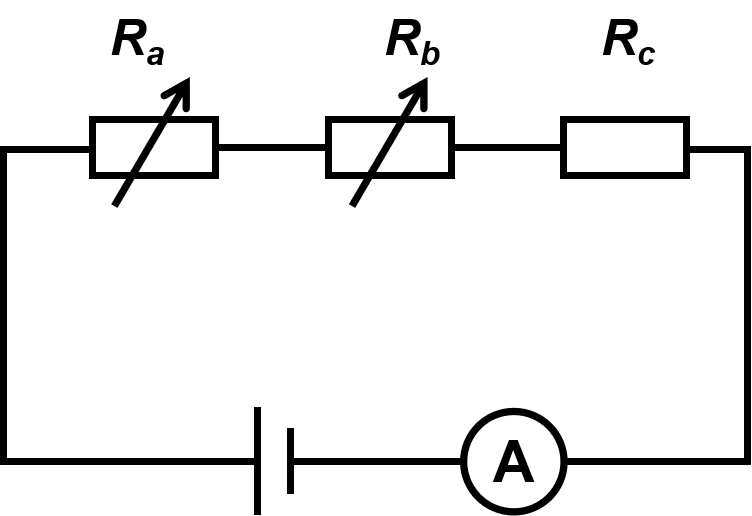


**Fig. S18** The equivalent circuit diagram of the rGOA-based pressure sensor


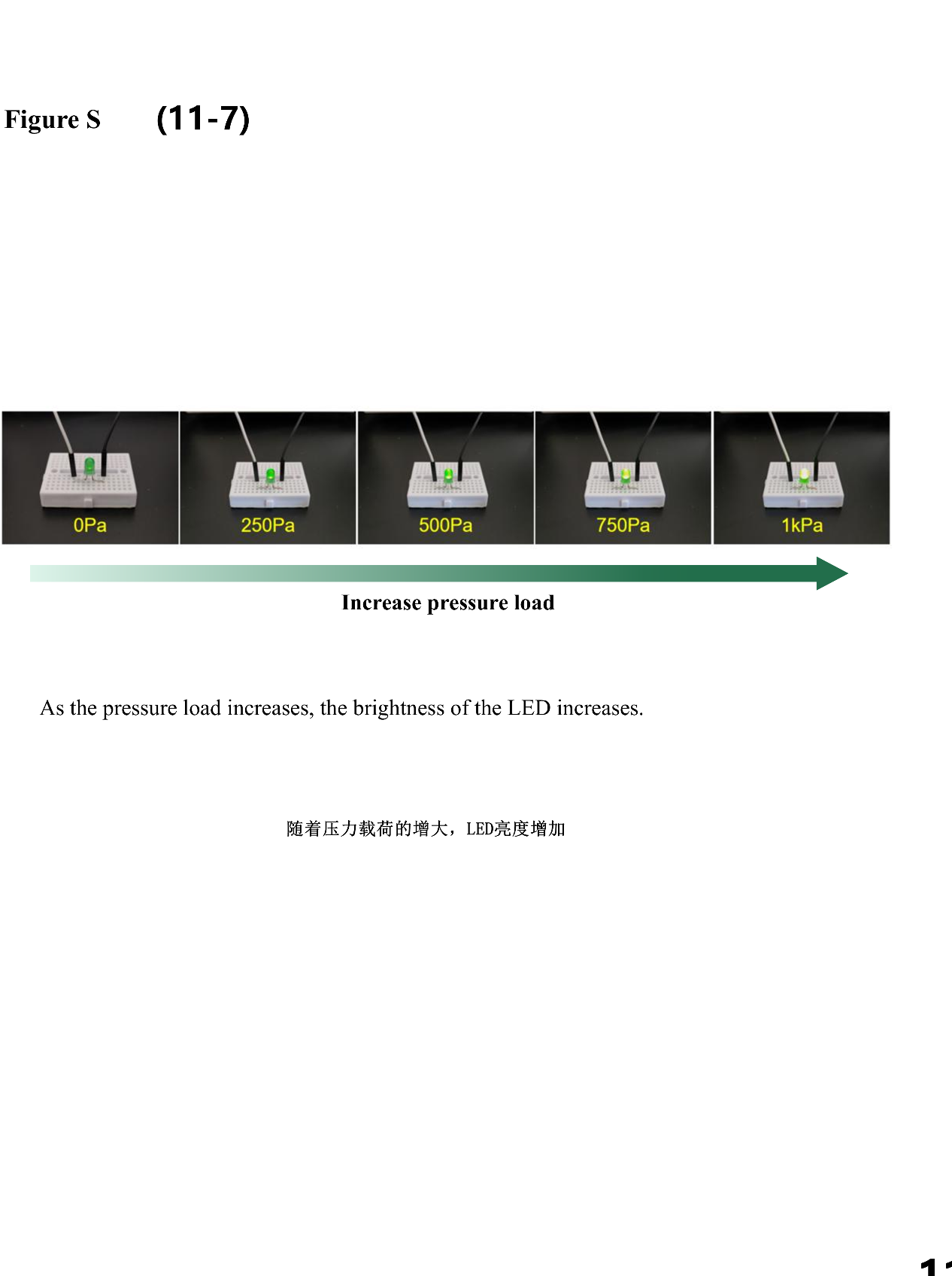


**Fig. S19** Photographs showing the increased brightness of the LED with the increasing pressure load


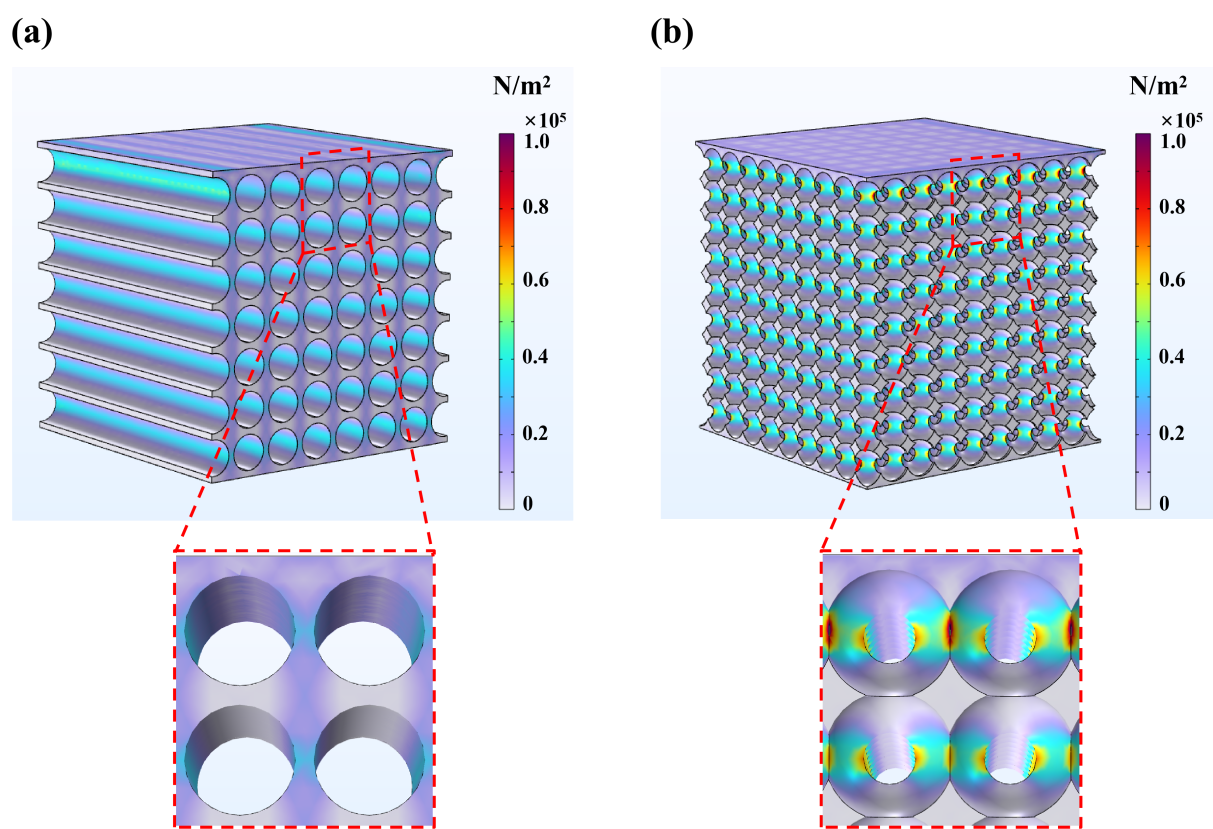


**Fig. S20** Comparison of simulated stress distribution between (**a**) anisotropic and (**b**) isotropic structures (with uniformly distributed spherical pores in the interior) under 5 kPa. The lower peak stress from the anisotropic structure indicates more uniform load distribution and reduced stress concentration


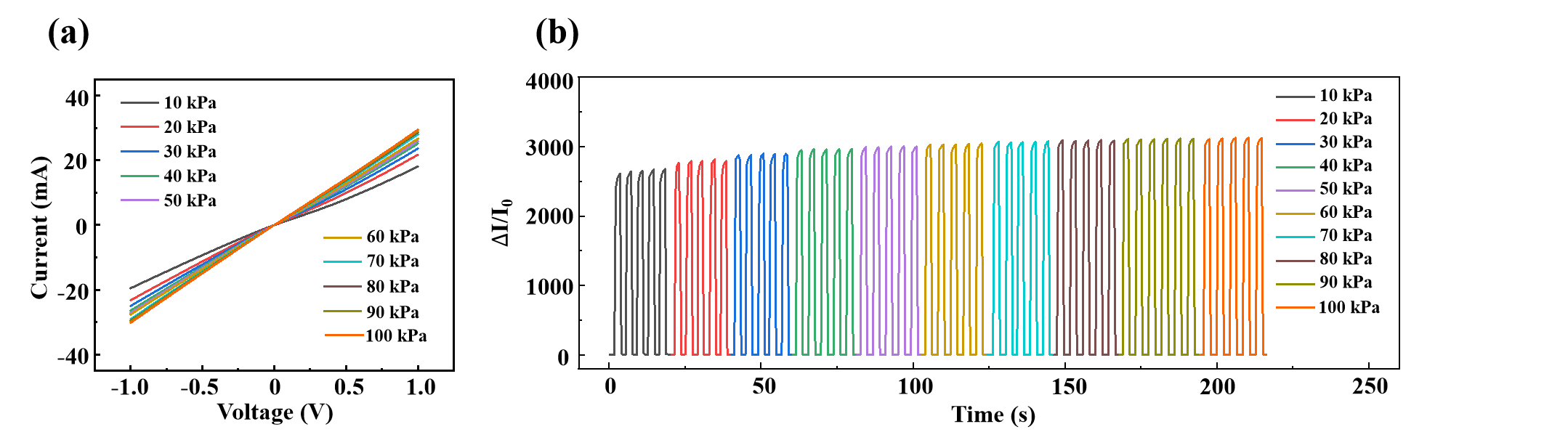


**Fig. S21** (**a**) I−V curves and (**b**) normalized relative current changes of the rGOA-based pressure sensor under varying pressure loads from 10 to 100 kPa


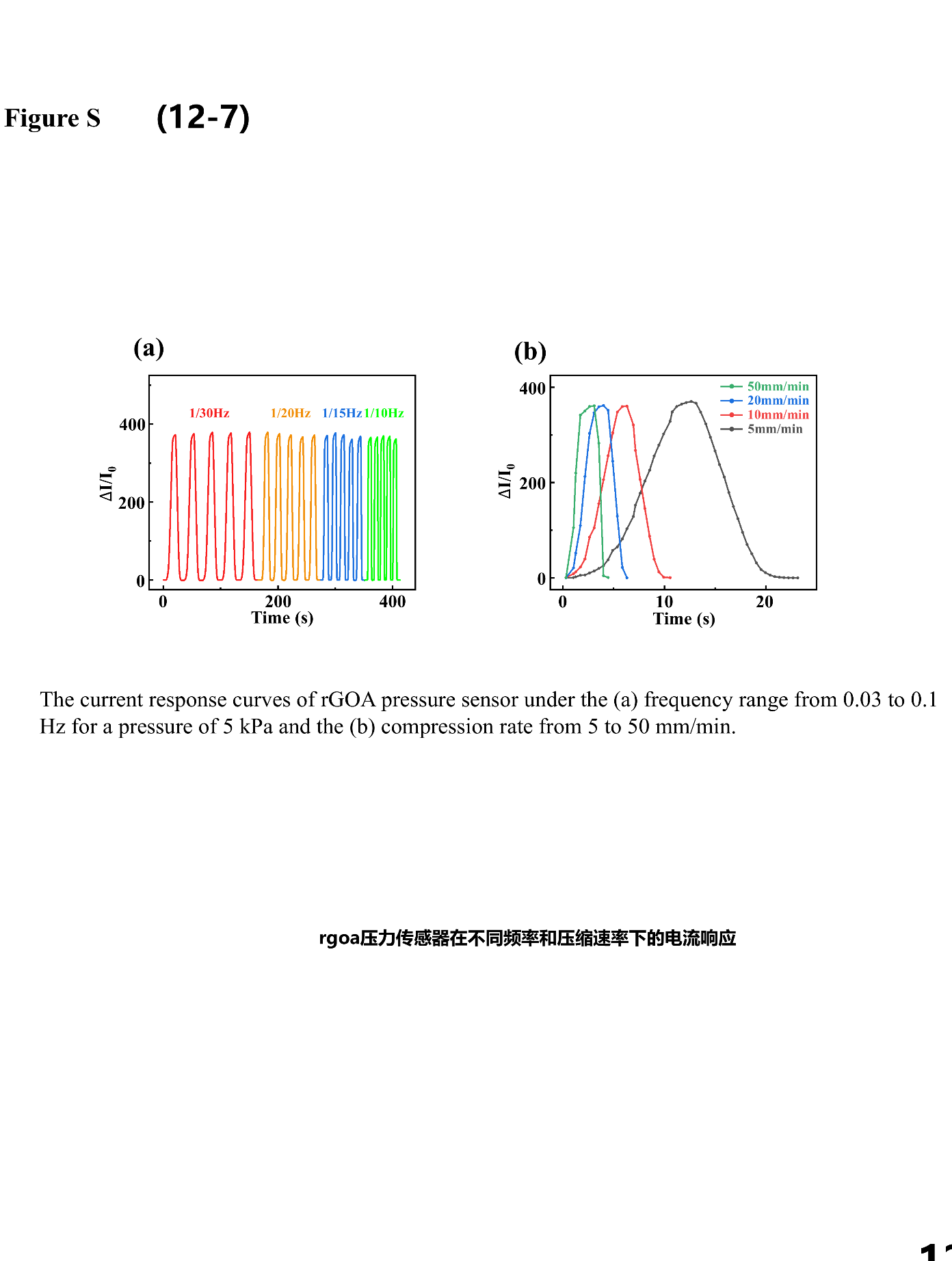


**Fig. S22** Comparison of the current response curves of the rGOA-based pressure sensor under varied (**a**) frequency from 0.03 to 0.1 Hz (at compression rates of 3, 5, 7, and 10 mm/min) and (**b**) compression rate from 5 to 50 mm/min to 5 kPa


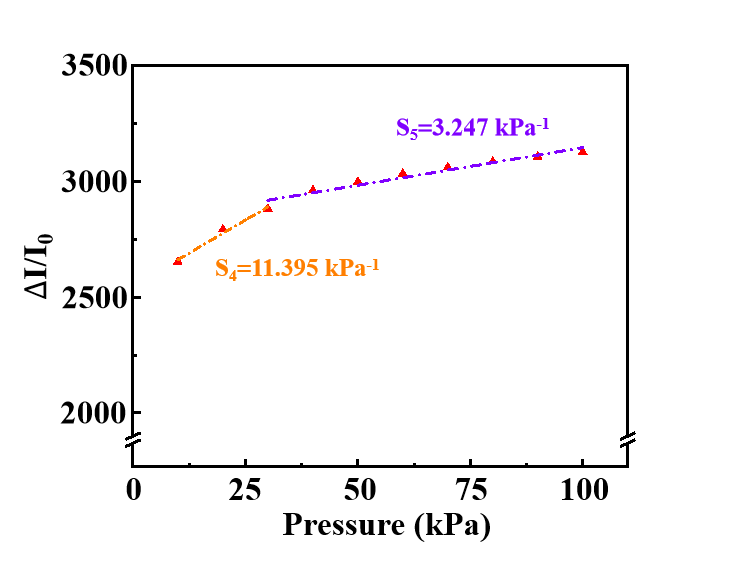


**Fig. S23** The sensitivity calibration curves of the rGOA-based pressure sensor under pressure loads ranging from 10 to 100 kPa


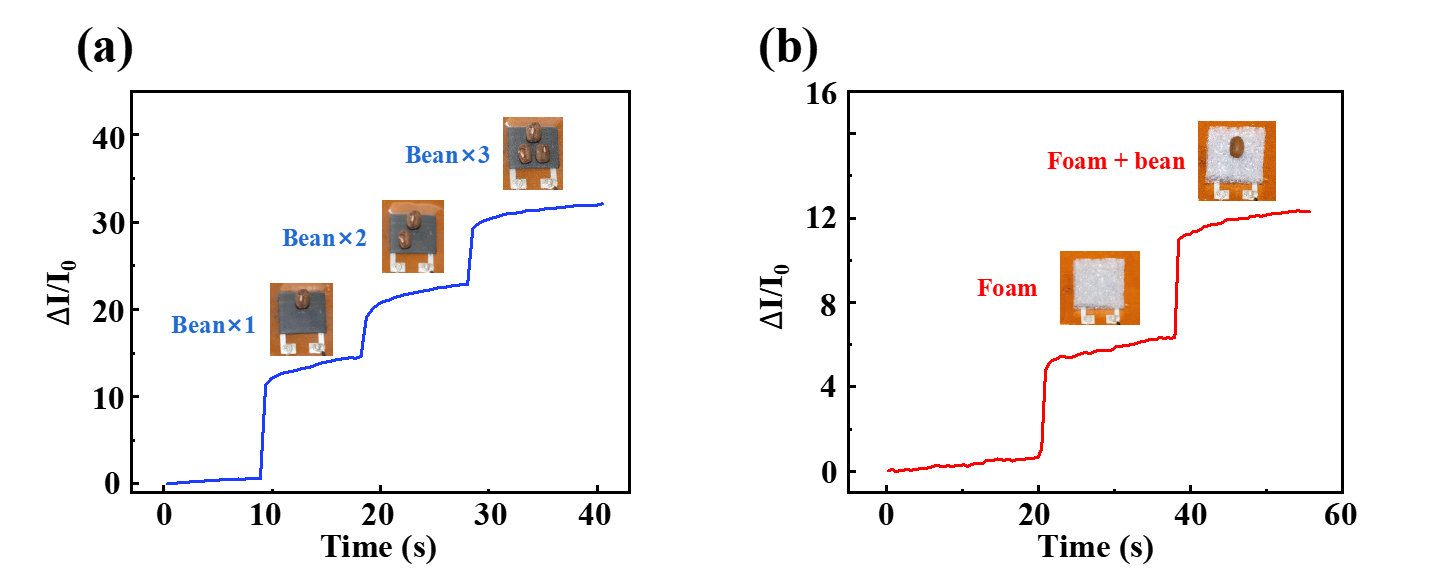


**Fig. S24** The response of the rGOA-based pressure sensor to tiny pressures from (**a**) one, two, and three beans, and (**b**) foam and bean

**
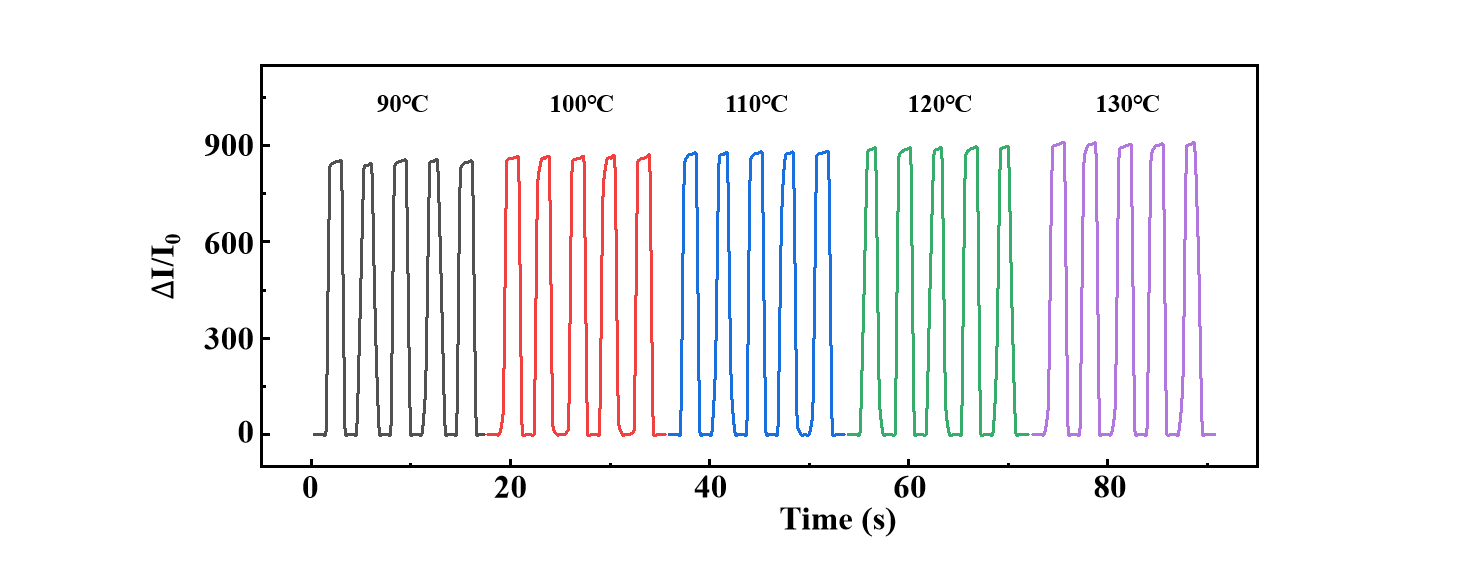
**

**Fig. S25** Current responses of the rGOA-based pressure sensor in the temperature range from 90 to 130℃

**
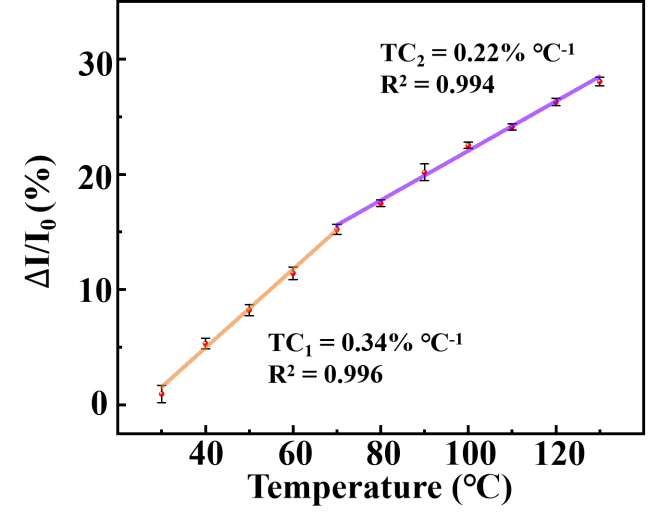
**

**Fig. S26** Temperature-dependent normalized current variation of the rGOA-based pressure sensor in the range from 30 to 130℃. Linear fit reveals two temperature coefficients of 0.34% ℃^-1^ (30-70℃) and 0.22% ℃^-1^ (70-130℃)

**
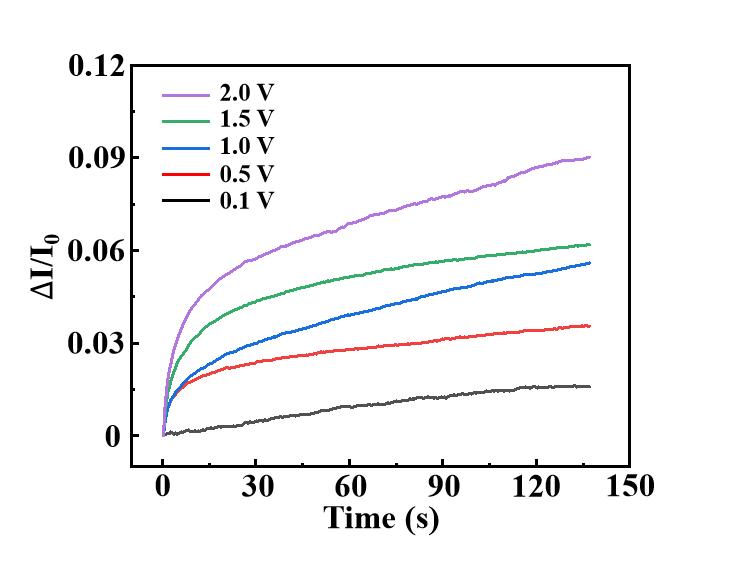
**

**Fig. S27** The current response of the rGOA-based pressure sensor under applied voltages ranging from 0.1 V to 2 V in the absence of an external load

**
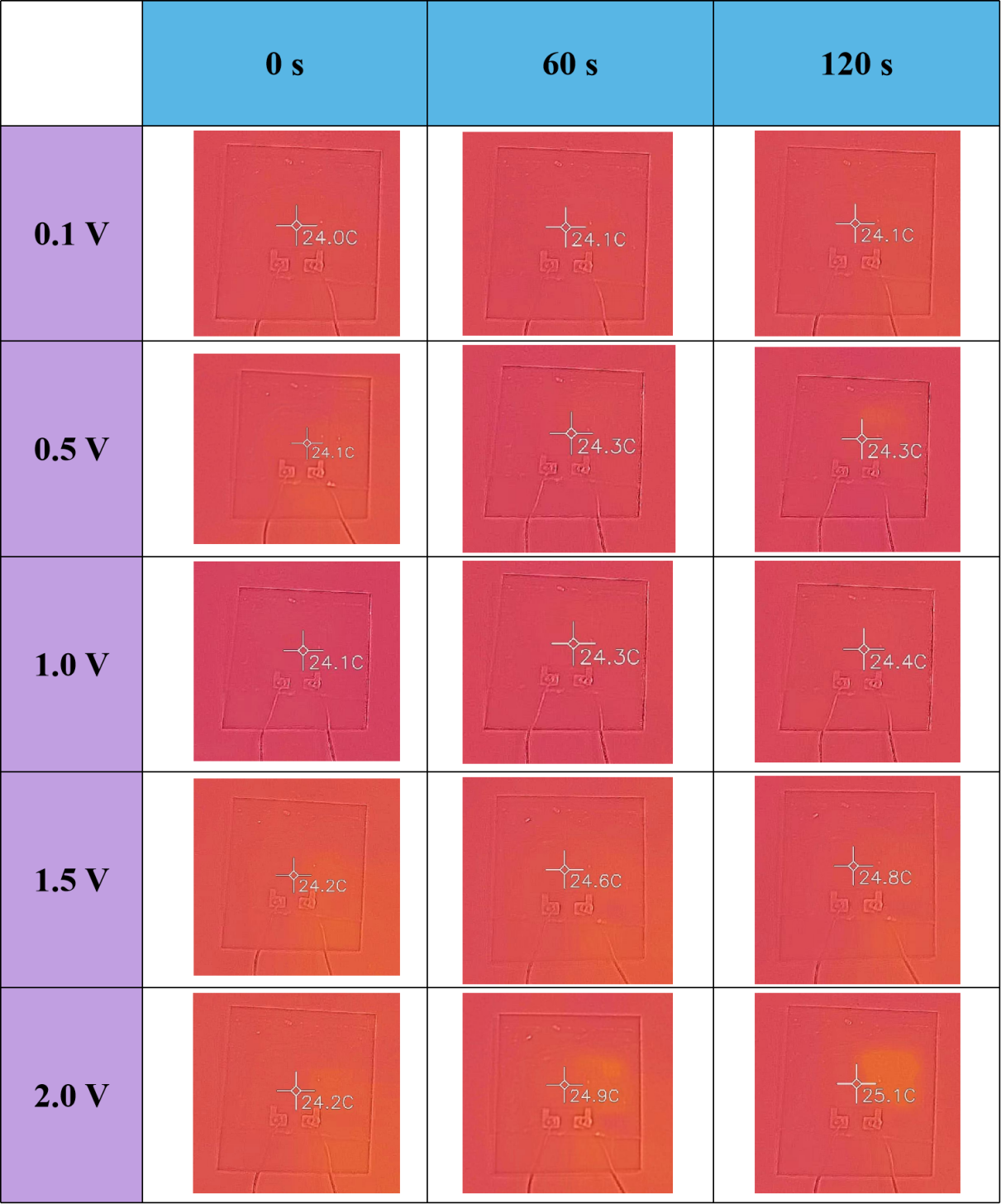
**

**Fig. S28** Infrared images of the rGOA-based pressure sensor showing temperature variation over time under applied voltages ranging from 0.1 V to 2 V

**
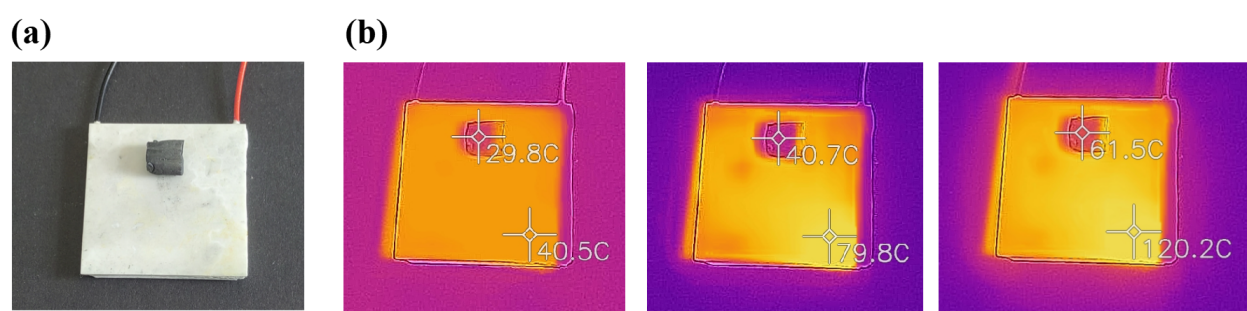
**

**Fig. S29** (**a**) The rGOA placed on the surface of Peltier and (**b**) the temperature difference was detected using an infrared thermometer


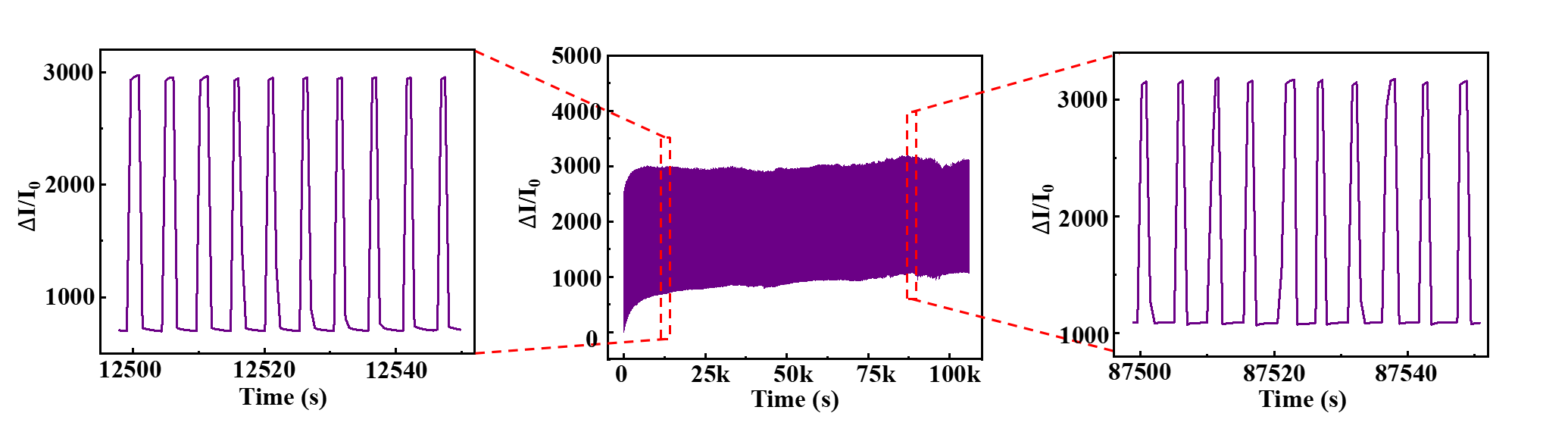


**Fig. S30** Repeatability test of the rGOA-based sensor over 20,000 loading/ unloading cycles (10 kPa), with the baseline drift increased by approximately 1.6× between 200 and 20,000 cycles


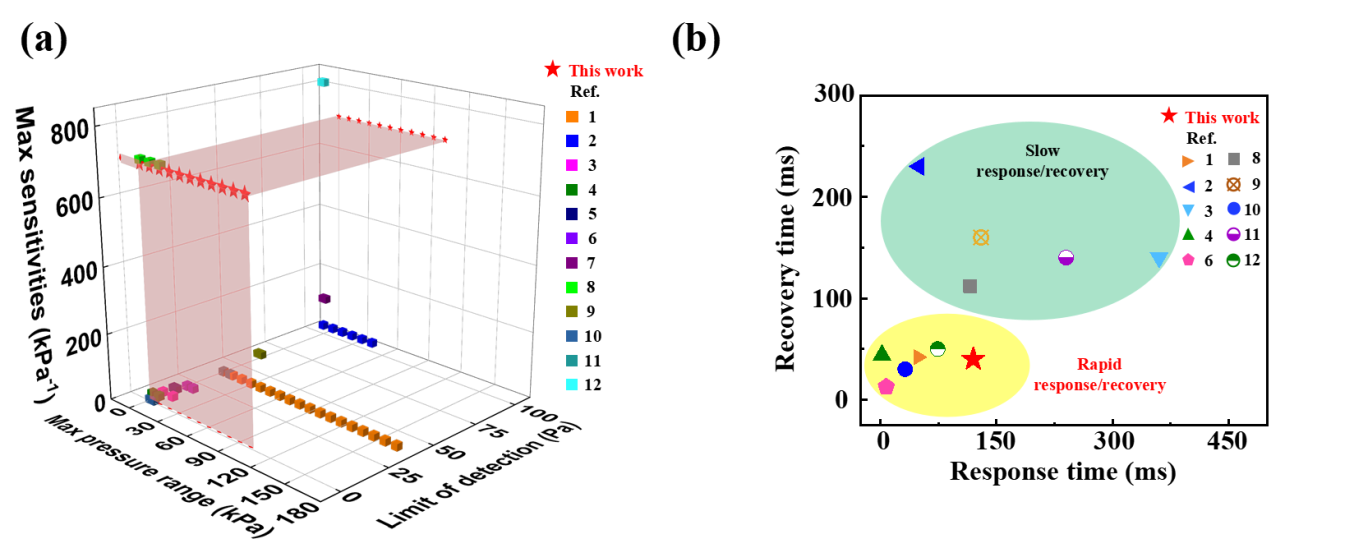


**Fig. S31** Comparison between the rGOA-based and other aerogel-based flexible pressure sensors in terms of (**a**) maximum sensitivity, limit of detection, maximum pressure range, and (**b**) response/recovery time


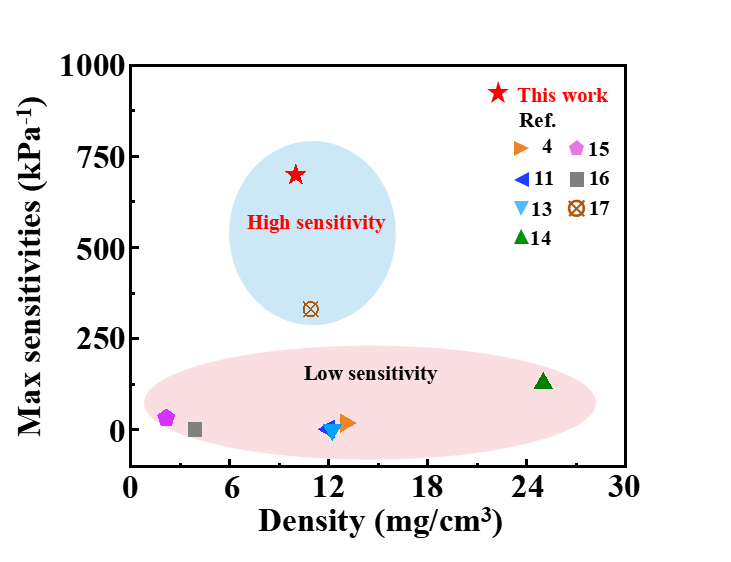


**Fig. S32** Comparison between the rGOA-based pressure sensor and other aerogel-based flexible pressure sensors in terms of maximum sensitivity and density


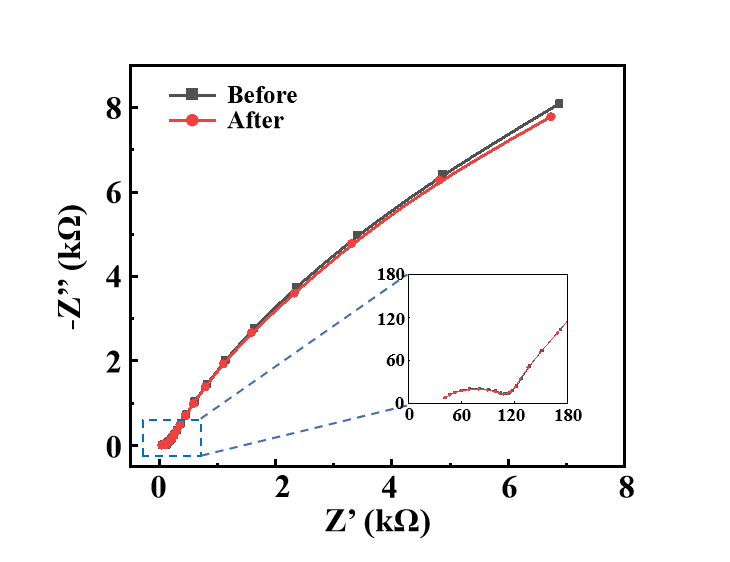


**Fig. S33** EIS spectra of the rGOA before and after 5,000 loading/unloading cycles to 250 Pa


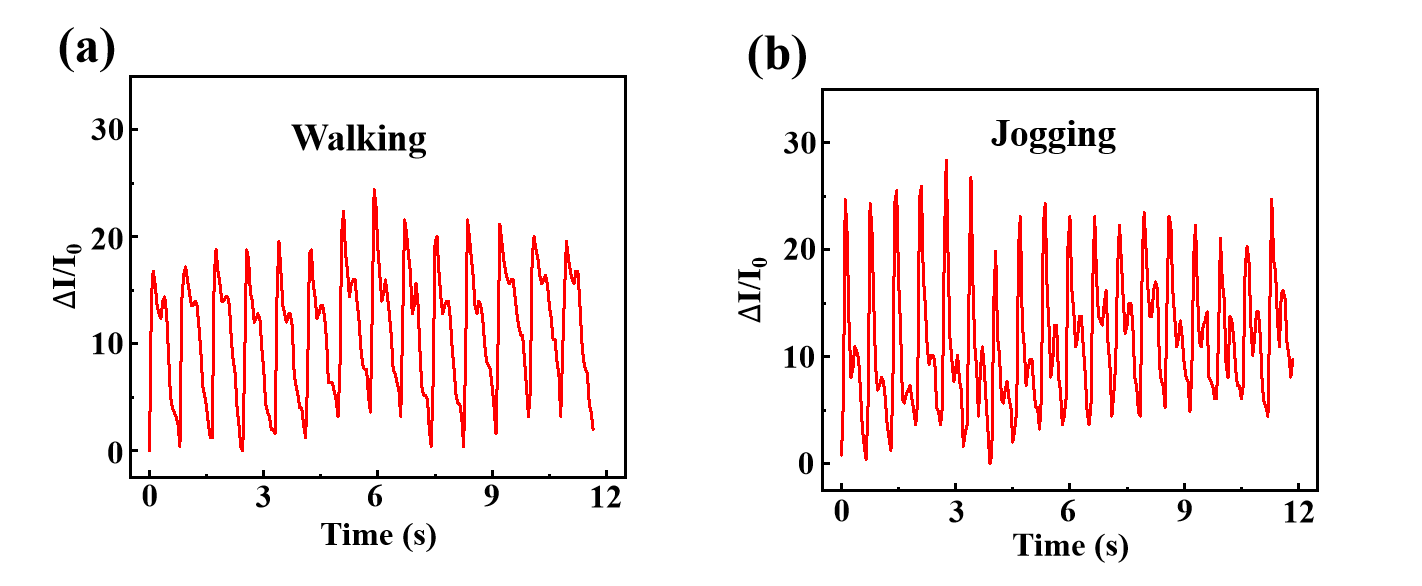


**Fig. S34** Periodic pulse signals measured from the radial artery of the wrist during (**a**) walking and (**b**) jogging


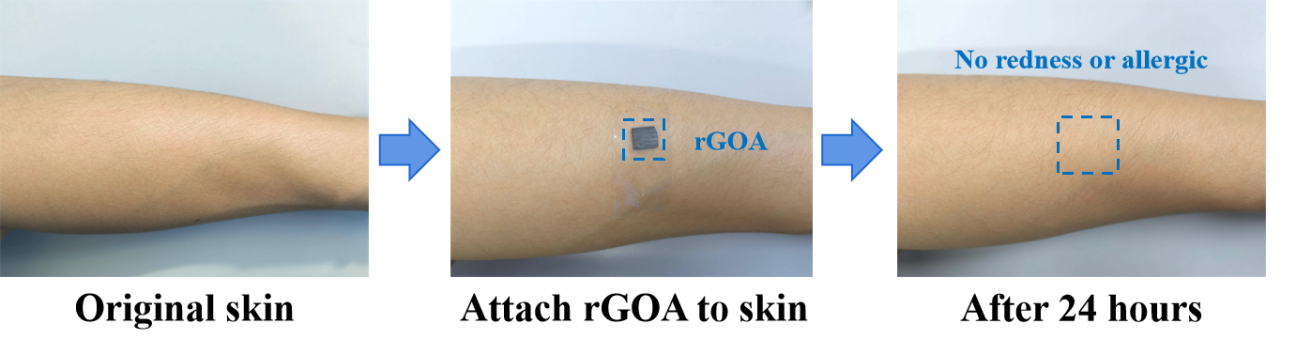


**Fig. S35** Optical images of the skin before and after attaching the rGOA (in the blue dashed box region) for 24 hours


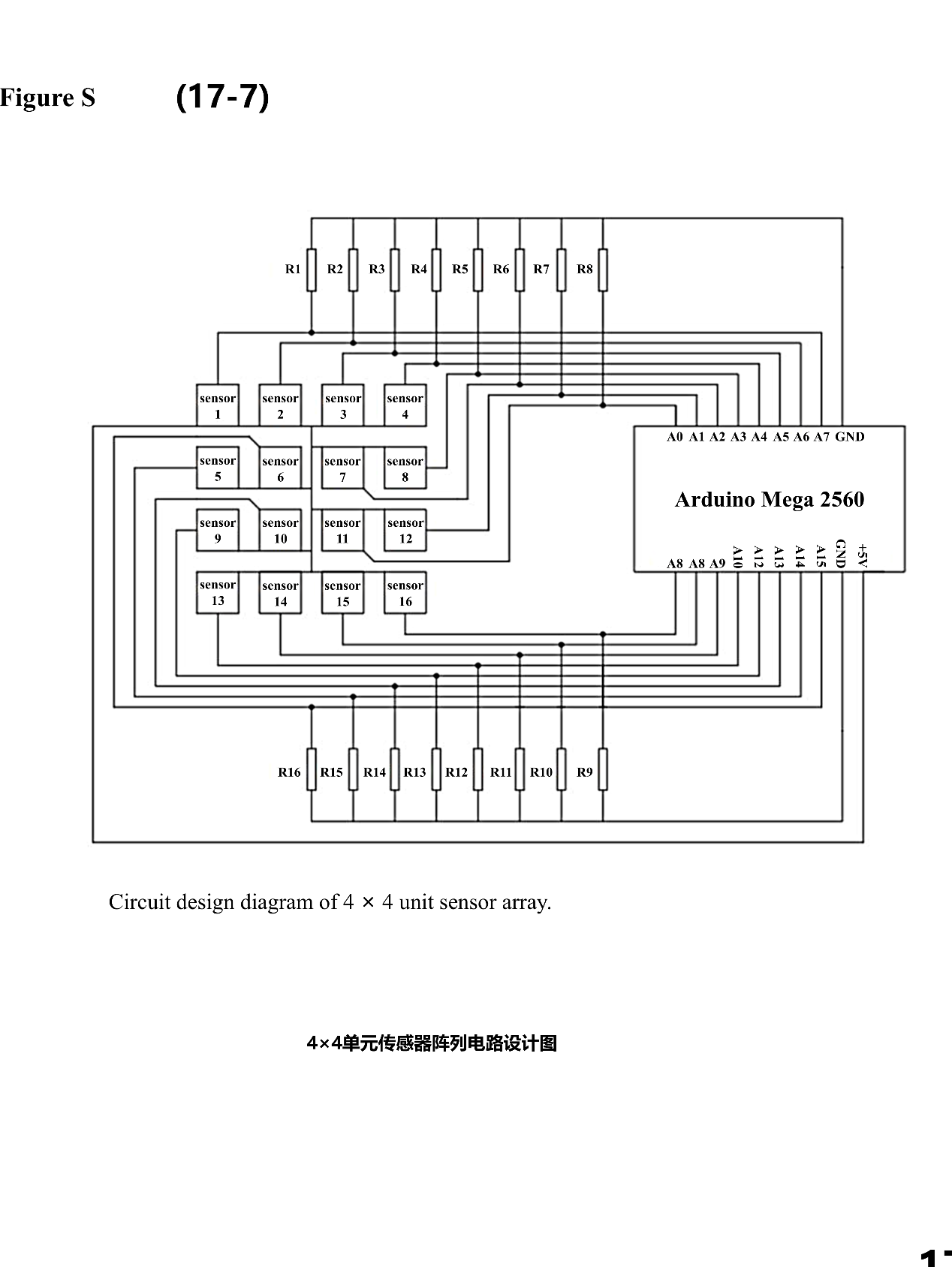


**Fig. S36** Circuit design diagram of the 4 × 4 pressure sensor array


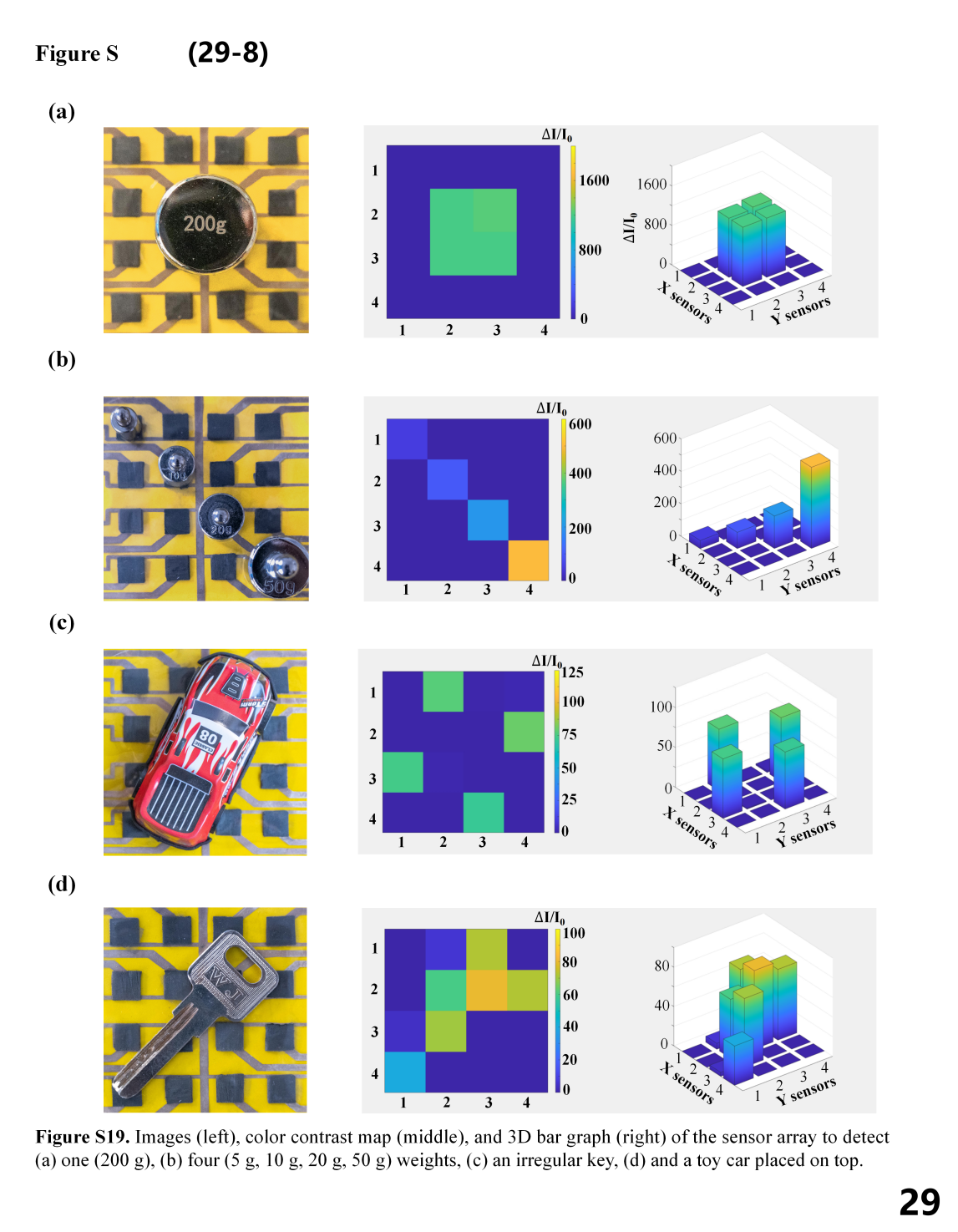


**Fig. S37** Images (left), color contrast map (middle), and 3D bar graph (right) of the sensor array to detect (**a**) one (200 g), (**b**) four (5 g, 10 g, 20 g, 50 g) weights, (**c**) an irregular key (**d**) and a toy car placed on top


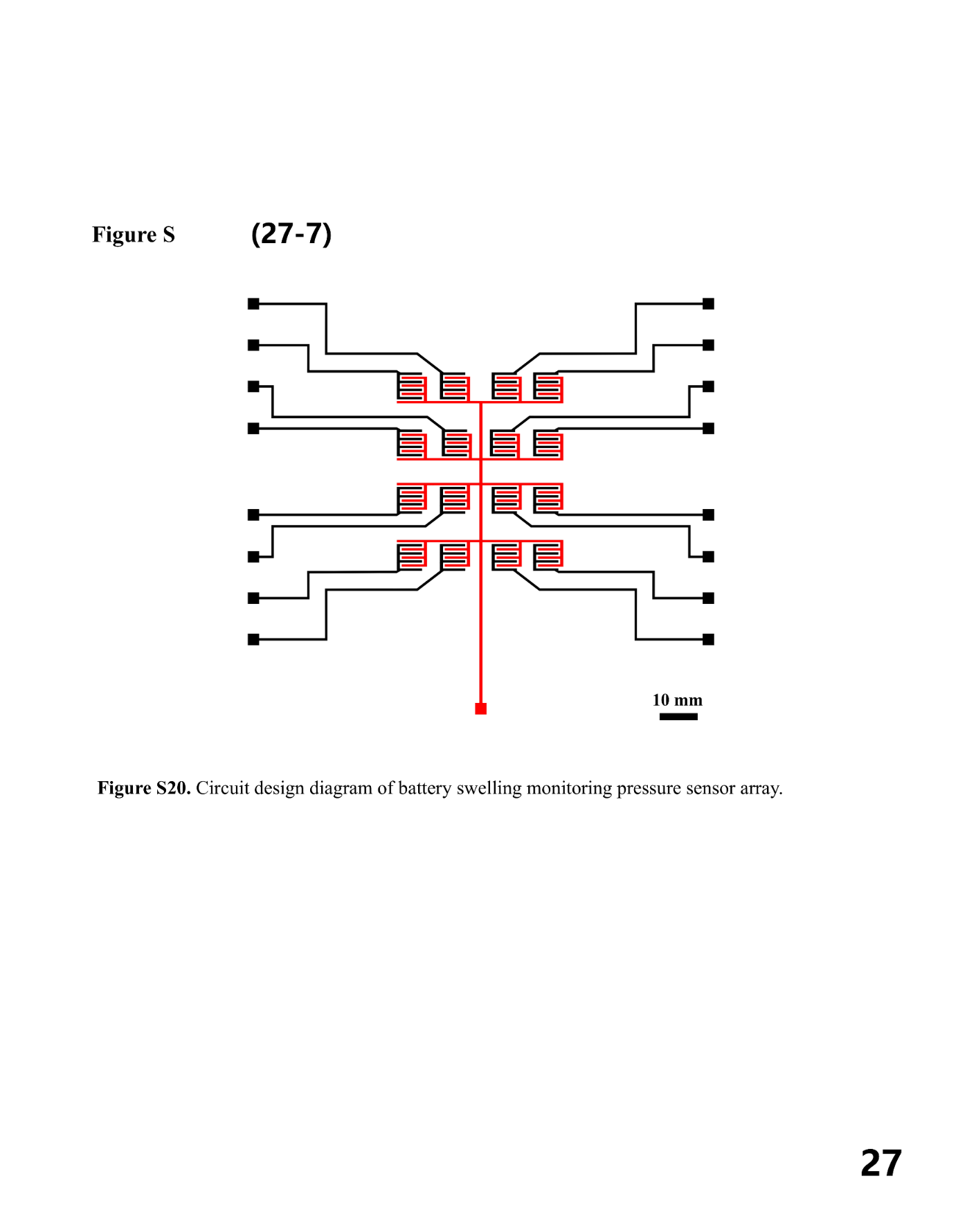


**Fig. S38** Circuit diagram of the pressure sensor array for battery monitoring


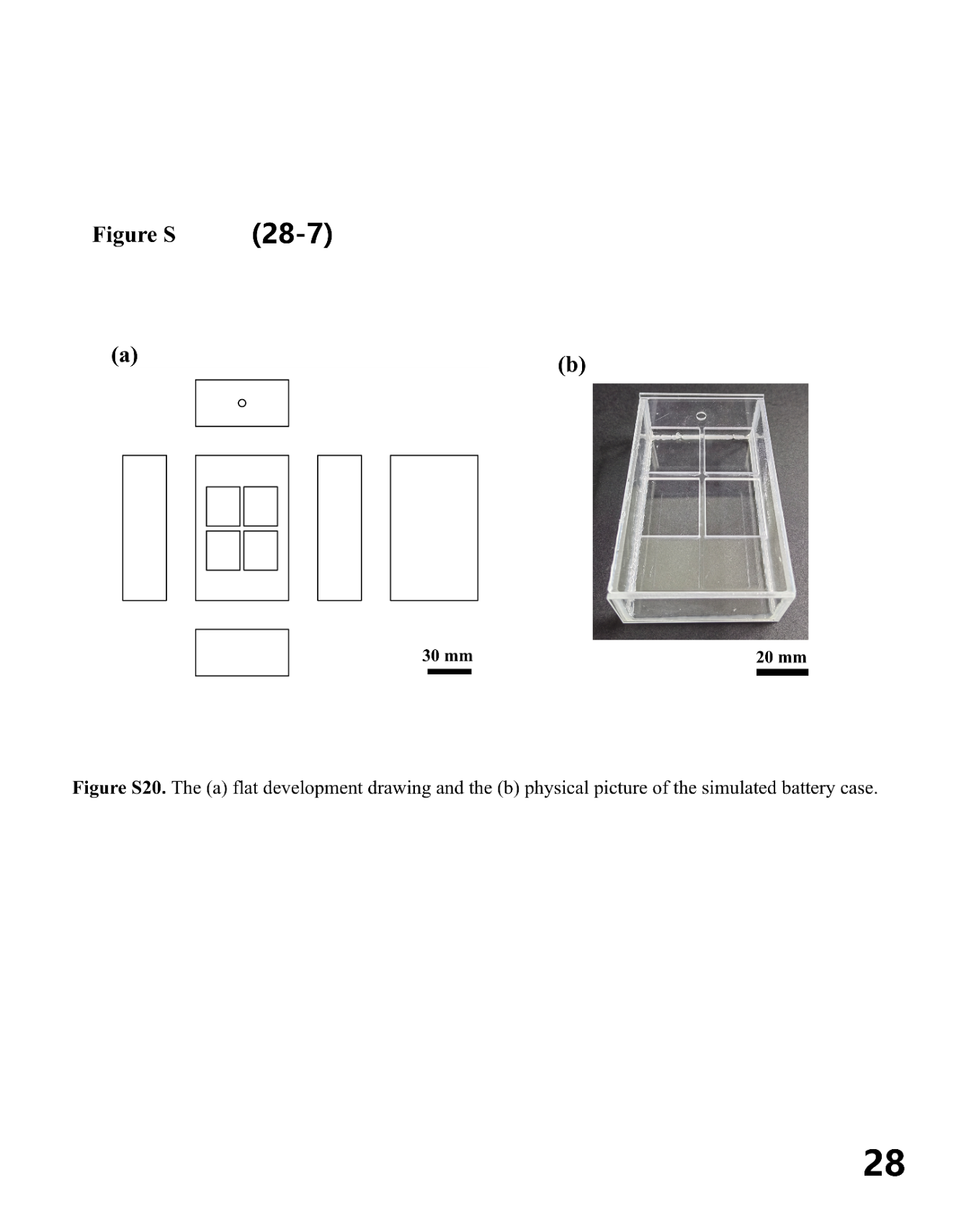


**Fig. S39** (**a**) Design and (**b**) photograph of the simulated battery case


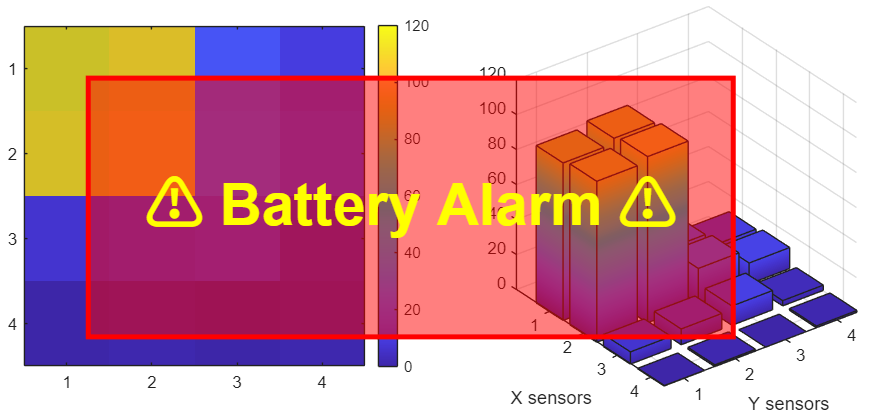


**Fig. S40** MATLAB-based alert interface activated upon reaching the warning threshold during battery swelling monitoring


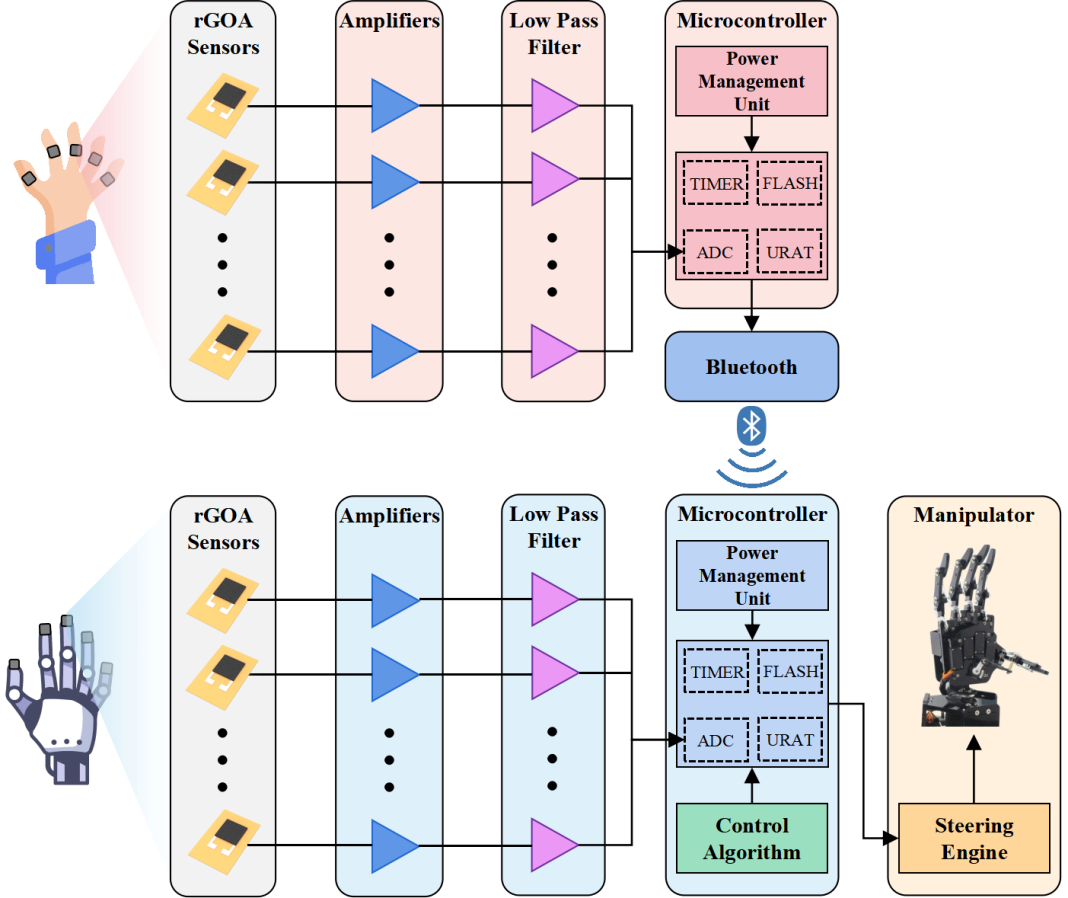


**Fig. S41** Schematic diagram showing the control circuit of the manipulator for teleoperation based on force feedback


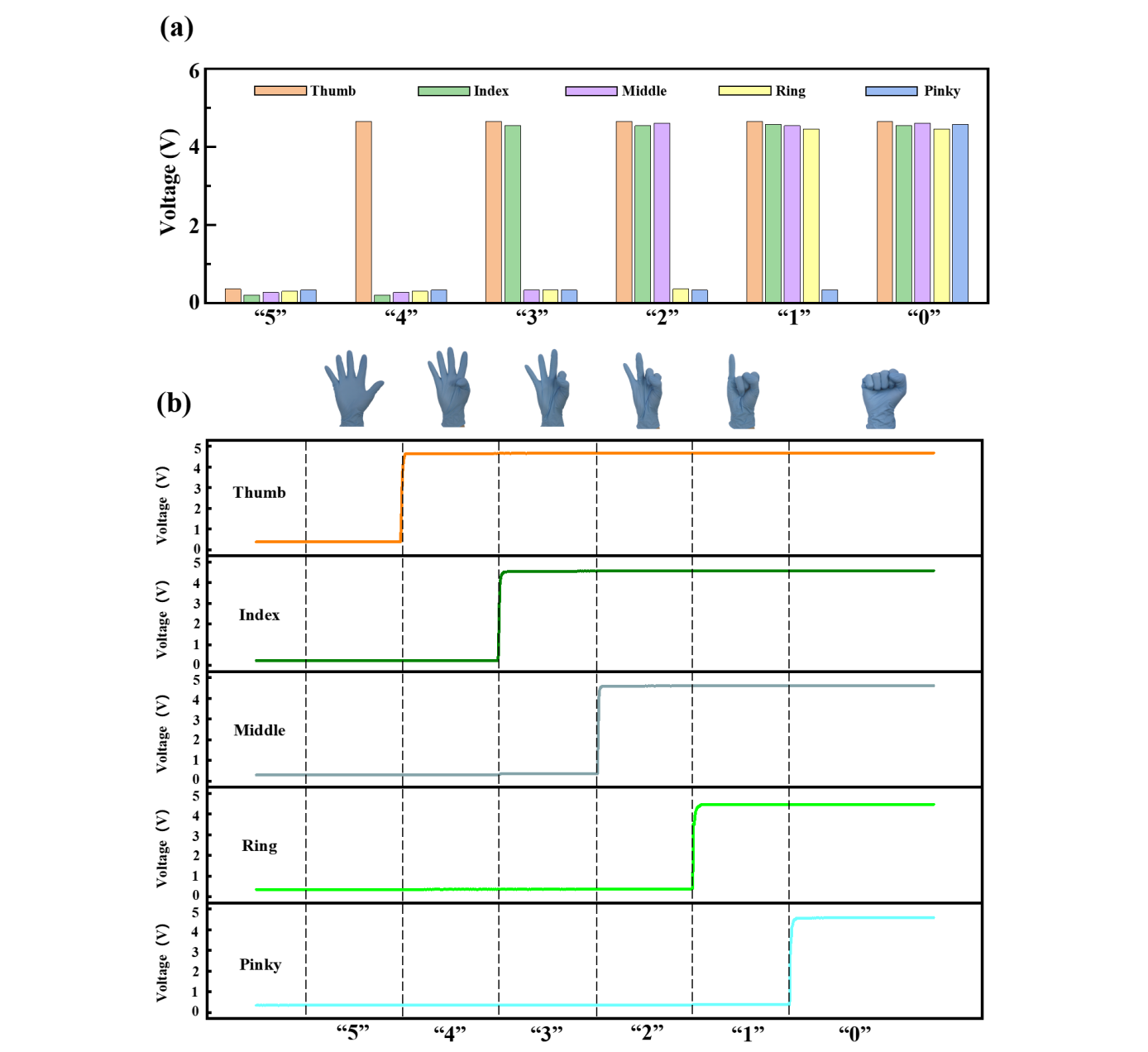


**Fig. S42** Voltage responses from the rGOA-based pressure sensors to six gestures: “5”, “4”, “3”, “2”, “1”, and “0”

**
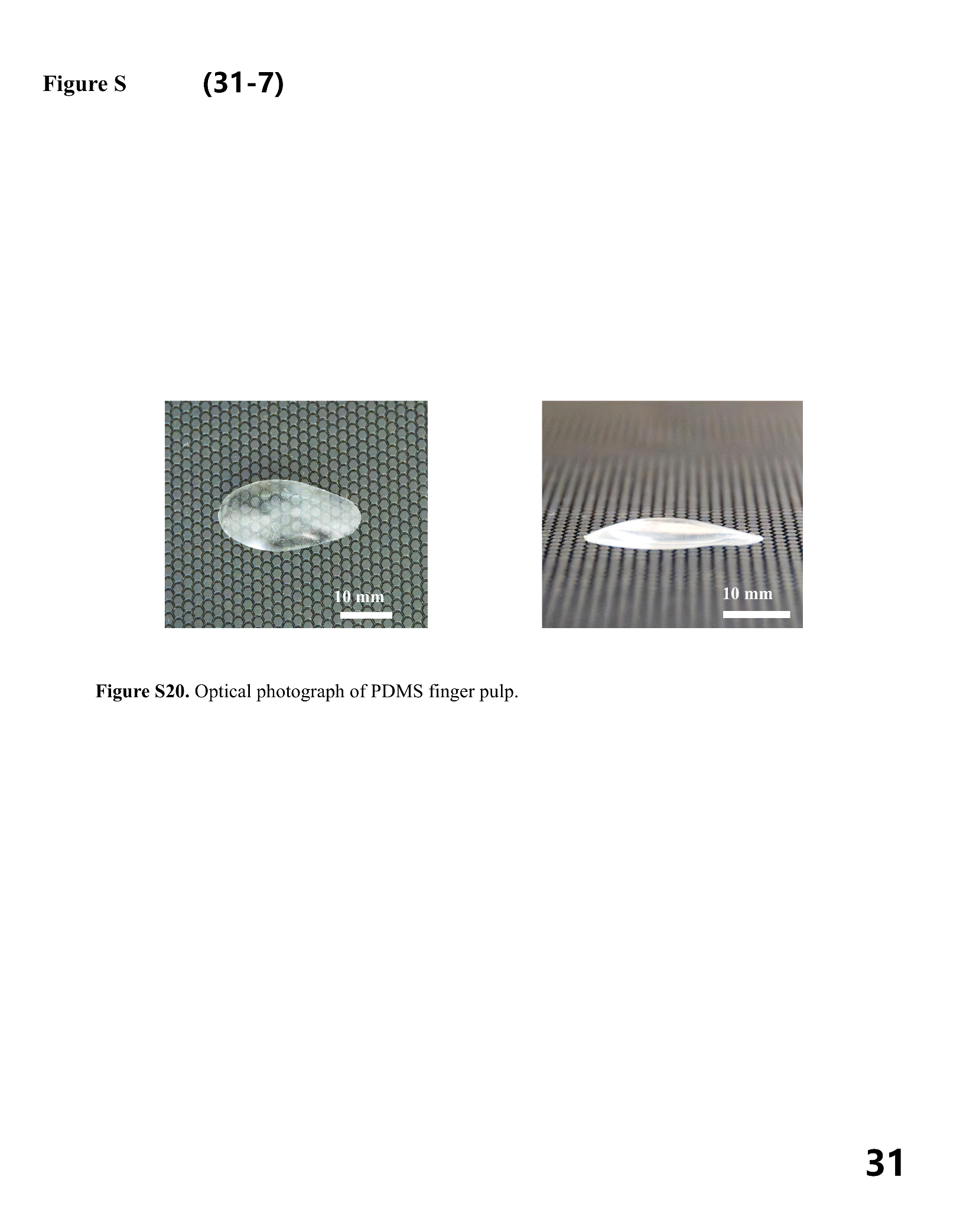
**

**Fig. S43** Photographs of the PDMS finger pulp

**
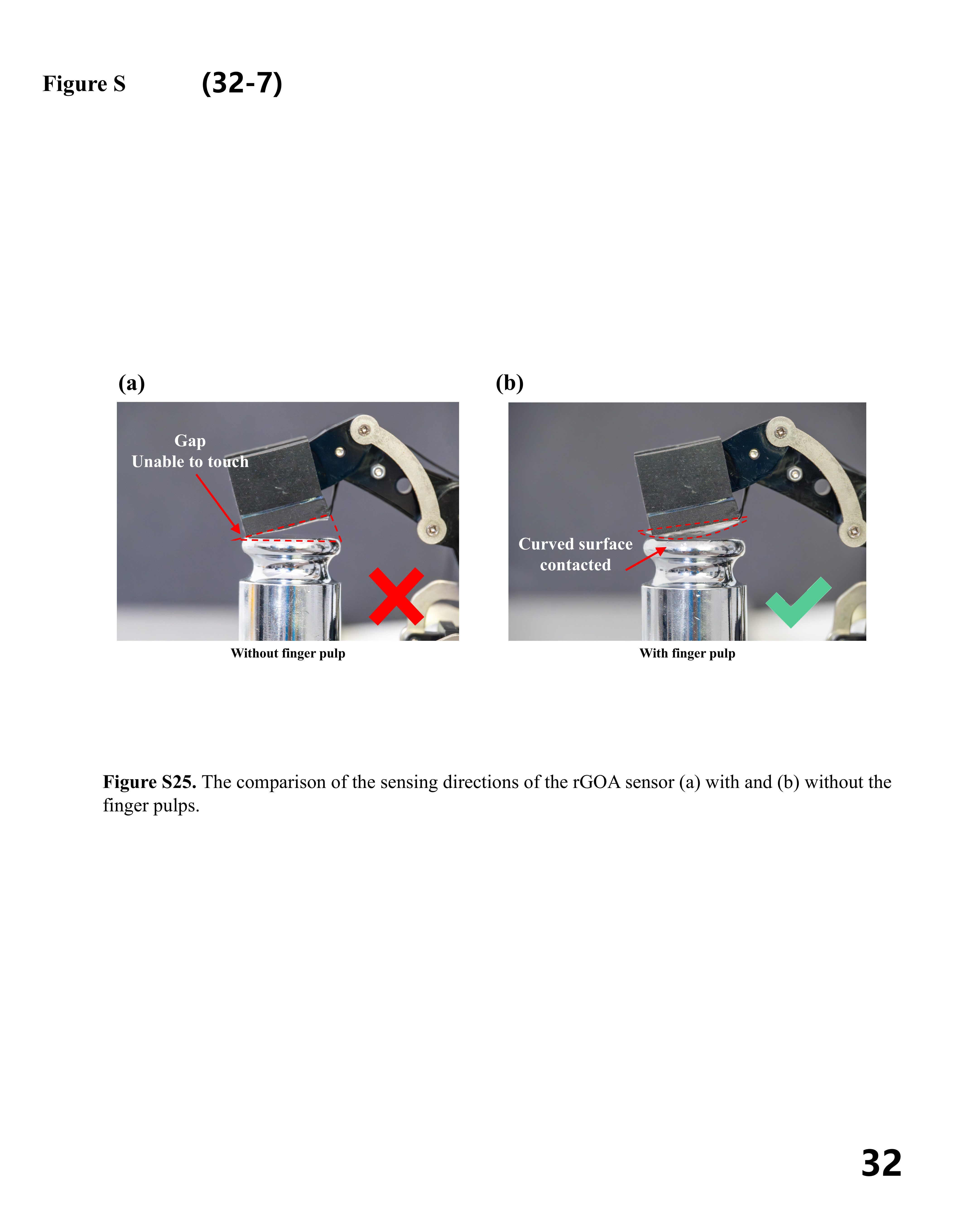
**

**Fig. S44** Comparison between the rGOA-based pressure sensors (**a**) without and (**b**) with the finger pulp


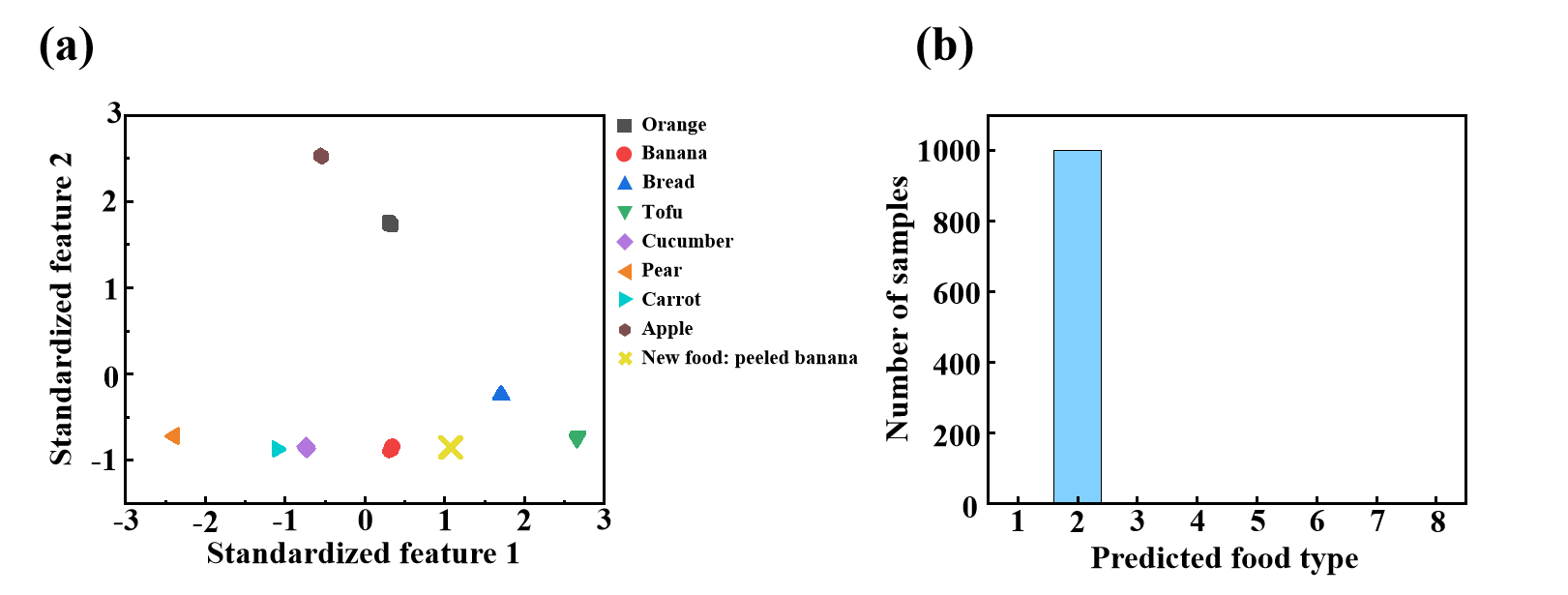


**Fig. S45** (**a**) Two-dimensional visualization of the eight trained food classes and the new unseen food sample of peeled banana in the feature space. Each trained class forms a well-separated cluster. (**b**) Prediction distribution of the new sample to banana

**Table S1** Performance comparison between the rGOA-based and other aerogel-based flexible pressure sensors

| **Aerogel materials** | **Sensitivity (kPa^-1^)** | **LOD (Pa)** | **Response/Recovery time (ms)** | **Cycling performance** | **Refs.** |
| --- | --- | --- | --- | --- | --- |
| Poly(ethylene glycol) diacrylate  /amino-functionalized carbon  nanotubes/MXene | 1.42 (0-0.2 kPa)  0.71 (0.12-8.69 kPa)  0.008 (8.69-164.15 kPa) | 40 | 49/42 | 3500 cycles  1 kPa | [S1] |
| Gradient graphene-aerogel | 19.9 (0.1-1 kPa)  16.3 (1-30 kPa)  29.6 (30-50 kPa) | 100 | 50/230 | 525 cycles  10 kPa | [S2] |
| Carbonized cotton aerogelelastomer hybrid porous material | 10.25 (0-1 kPa)  8.89 (1-10.53 kPa) | 7.3 | 360/140 | 10000 cycles  50% compression | [S3] |
| Cotton-like 3D graphene fiber aerogel | 18.55 (0-0.38 kPa)  2.73 (0.38-4 kPa)  0.42 (4-7 kPa) | 2 | 2/44 | 3000 cycles  50% compression | [S4] |
| Reduced graphene  oxide cross-linked by sustainable-macromolecule-derived carbon | 10.2 (0.75-2.75 kPa) | 13 | -/- | 1000 cycles  2.75 kPa | [S5] |
| Cellulose sub-micron fibers/carbon black | 0.234 (0-2 kPa)  0.101 (2-3.8 kPa)  0.047 (3.8-6.5 kPa) | 20 | 7.7/12.8 | 1000 cycles  1.5 kPa | [S6] |
| Anisotropic cross-linked chitosan  and reduced graphene oxide | 9.23 (0-0.5 kPa)  21.36 (0.5-1 kPa)  38.76 (1-2.5 kPa) | 100 | 60/- | 20000 cycles  50% compression | [S7] |
| 3D chitosan/MXene | 709.38 (0-1 kPa)  252.37 (1-20 kPa) | 1.41 | 116/112 | 10000 cycles  1 kPa | [S8] |
| Cellulose nanofiber/graphene oxide/acrylonitrile  butadiene styrene-derived carbon aerogel | 17.65 (0-0.36 kPa)  3.4 (1.5-3.2 kPa) | 60 | 130/160 | 500 cycles  60% compression | [S9] |
| Tannic acid/cellulose fibers | 4 (0-0.15 kPa)  1.6 (0.15-1 kPa)  0.6 (1-4 kPa) | 0.8 | 32/30 | 3000 cycles  0.5 kPa | [S10] |
| Graphene/aramid nanofiber/  polyaniline nanotube | 1.73 (0-0.7 kPa)  0.3 (0.7-7 kPa) | 40 | 240/140 | 3000 cycles  10% compression | [S11] |
| Cellulose nanofiber/carbon  nanotube/MXene | 817.3 (0-0.2 kPa)  234.9 (0.2-1.5 kPa) | 100 | 74/50 | 2000 cycles  30% compression | [S12] |
| Graphene oxide/dopamine/polyaniline/nitrogen-doped | 0.10 (0-11.58 kPa) | 0 | -/- | 150 cycles  45% compression | [S13] |
| 1D nanofiber-reinforced 2D pyrrole-reduced graphene oxide | 32.39 (0-30.5 kPa)  12.36 (30.5-65.3 kPa) | - | 280/50 | 10000 cycles  20% compression | [S14] |
| Graphene oxide/bacterial cellulose | 51.4 (0-3 kPa)  27.8 (3-12 kPa)  5.4 (12-30 kPa) | 20 | ~300/~300 | 200 cycles  18 kPa | [S15] |
| Graphene-multiwalled carbon nanotubes/polyurethane | 0.1 (0-10 kPa)  0.0035 (10-50 kPa) | 200 | 720/770 | 1000 cycles  10 kPa | [S16] |
| Reduced graphene oxide aerogel  (rGOA) | **609.960 (0-1 kPa)**  **238.117 (1-3 kPa)**  **132.301 (7-10 kPa)**  **11.395 (10-30 kPa)**  **3.247 (30-100 kPa)** | **1** | **120/40** | **20000 cycles**  **10 kPa** | **This work** |

**Table S2** Comparison of the density and max sensitivity between rGOA-based and other aerogel-based flexible pressure sensors.

| Density (mg/cm^3^) | Max sensitivity (kPa^-1^) | Refs. |
| --- | --- | --- |
| 13 | 18.55 | [S4] |
| 12 | 1.73 | [S11] |
| 12.2 | 0.1 | [S13] |
| 25 | 128 | [S17] |
| 2.14 | 32.85 | [S18] |
| 3.88 | 0.83 | [S19] |
| 10.9 | 331 | [S20] |
| **10** | **698.96** | **This work** |

**Table S3** Performance comparison between the rGOA-based and other flexible piezoresistive sensors

| **Materials** | **Sensitivity (kPa^-1^)** | **LOD (Pa)** | **Response/Recovery time (ms)** | **Cycling performance** | **Refs.** |
| --- | --- | --- | --- | --- | --- |
| MXene/cellulose nanofibers (CNFs) | 6.023 (0.11-8.267 kPa)  21.457 (8.267-11.022 kPa)  2.223 (11.022-110.22 kPa) | 110 | 41.84/20.82 | 6000 cycles  16.534 kPa | [S21] |
| MXene/dot-structured cellulose paper | 39.58 (0-1.01 kPa)  11.95 (1.01-60 kPa) | 2.8 | 93/69 | 10000 cycles  5 kPa | [S22] |
| MXene/cotton soft tissue | 36.87 (0-1.3 kPa)  11.39 (1.3-60 kPa)  1.25 (60-79 kPa) | 3.7 | 113/81 | 10000 cycles  1.45 kPa | [S23] |
| MXene/CNFs | 145.5 (0-18 kPa)  25.7 (18-50 kPa) | 320 | 68/40 | 10000 cycles  35.71 kPa | [S24] |
| Polydimethylsiloxane (PDMS)/carbon nanotube (CNT)/thermoplastic polyurethane /MXene | 82.17 (0-25 kPa) | - | 100/100 | 2000 cycles  ~10 kPa | [S25] |
| Ag@waffle-structured MXene | 3.04 (0-120 kPa)  1.52 (120-180 kPa)  0.365 (180-300 kPa) | - | 45/30 | 2000 cycles  210 kPa | [S26] |
| CNTs/carbon black/PDMS conductive sponge | 7.10 (0-25 kPa)  2.96 (25-135 kPa)  1.09 (135-350 kPa) | 10 | 175/165 | 2000 cycles  ~30 kPa | [S27] |
| CNTs/graphene nanoparticles /PDMS | 38.28 (0-1.5 kPa)  5.75 (1.5-5.8 kPa)  0.79 (5.8-20 kPa) | - | 100/200 | 2000 cycles  ~5 kPa | [S28] |
| Reduced graphene oxide aerogel  (rGOA) | **609.960 (0-1 kPa)**  **238.117 (1-3 kPa)**  **132.301 (7-10 kPa)**  **11.395 (10-30 kPa)**  **3.247 (30-100 kPa)** | **1** | **120/40** | **20000 cycles**  **10 kPa** | **This work** |

**Supplementary References**

1. J. Huang, H. Liu, Q. Chen, H. Xie, S. Zhou, Shape-programmable lamellar aerogel enabling 3D wireless point-of-care electronics for assistance in pressure injury prevention. Adv. Funct. Mater. **35**(13), 2418037 (2025). <https://doi.org/10.1002/adfm.202418037>
2. J. Liu, W. Li, J. Li, K. Wang, Y. Wen et al., Graphene aerogel-based pressure sensors with gradient structure for subtle physiological signals and motion monitoring. Adv. Mater. Technol. **9**(10), 2302169 (2024). <https://doi.org/10.1002/admt.202302169>
3. Y. Liu, Z. Zhong, C. Liang, F. Wang, H. Xu et al., Highly compressive aerogel-elastomer hybrid porous material with redistributed strain for high-sensitive soft pressure sensors. Chem. Eng. J. **496**, 154069 (2024). <https://doi.org/10.1016/j.cej.2024.154069>
4. T. Yang, C. Ma, C. Lin, J. Wang, W. Qiao et al., Innovative fabrication of ultrasensitive and durable graphene fiber aerogel for flexible pressure sensors. Carbon **229**, 119484 (2024). <https://doi.org/10.1016/j.carbon.2024.119484>
5. Z. Zeng, N. Wu, W. Yang, H. Xu, Y. Liao et al., Sustainable-macromolecule-assisted preparation of cross-linked, ultralight, flexible graphene aerogel sensors toward low-frequency strain/pressure to high-frequency vibration sensing. Small **18**(24), 2202047 (2022). <https://doi.org/10.1002/smll.202202047>
6. Y. Zhang, P. Zhu, H. Sun, X. Sun, Y. Ye et al., Superelastic cellulose sub-micron fibers/carbon black aerogel for highly sensitive pressure sensing. Small **20**(13), e2310038 (2024). <https://doi.org/10.1002/smll.202310038>
7. Y. Wang, Z. Qin, D. Wang, D. Liu, Z. Wang et al., Microstructure-reconfigured graphene oxide aerogel metamaterials for ultrarobust directional sensing at human–machine interfaces. Nano Lett. **24**(38), 12000–12009 (2024). <https://doi.org/10.1021/acs.nanolett.4c03706>
8. C. Shang, X. He, X. Li, Z. Liu, Y. Song et al., One 3D aerogel wearable pressure sensor with ultrahigh sensitivity, wide working range, low detection limit for voice recognition and physiological signal monitoring. Sci. China Mater. **66**(5), 1911–1922 (2023). <https://doi.org/10.1007/s40843-022-2307-6>
9. D. Liao, Y. Wang, P. Xie, C. Zhang, M. Li et al., A resilient and lightweight cellulose/graphene oxide/polymer-derived multifunctional carbon aerogel generated from Pickering emulsion toward a wearable pressure sensor. J. Colloid Interface Sci. **628**(Pt A), 574–587 (2022). <https://doi.org/10.1016/j.jcis.2022.07.188>
10. G. Wang, X. Liu, Z. Song, D. Yu, G. Li et al., Multifunctional flexible pressure sensor based on a cellulose fiber-derived hierarchical carbon aerogel. ACS Appl. Electron. Mater. **5**(3), 1581–1591 (2023).
11. Y. Zou, Z. Chen, X. Guo, Z. Peng, C. Yu et al., Mechanically robust and elastic graphene/aramid nanofiber/polyaniline nanotube aerogels for pressure sensors. ACS Appl. Mater. Interfaces **14**(15), 17858–17868 (2022). <https://doi.org/10.1021/acsami.2c02538>
12. T. Xu, Q. Song, K. Liu, H. Liu, J. Pan et al., Nanocellulose-assisted construction of multifunctional MXene-based aerogels with engineering biomimetic texture for pressure sensor and compressible electrode. Nano-Micro Lett. **15**(1), 98 (2023). <https://doi.org/10.1007/s40820-023-01073-x>
13. Z. Deng, C. Gao, S. Feng, H. Zhang, Y. Liu et al., Highly Compressible, Light-Weight and robust Nitrogen-Doped graphene composite aerogel for sensitive pressure sensors. Chem. Eng. J. **471**, 144790 (2023). <https://doi.org/10.1016/j.cej.2023.144790>
14. Z. Qin, Z. Wang, D. Li, B. Zhao, Y. Lv et al., Lightweight nanofiber-reinforced pyrrole-reduced graphene oxide aerogel for pressure sensor and oil/water separation material. Adv. Mater. Technol. **8**(23), 2300739 (2023). <https://doi.org/10.1002/admt.202300739>
15. J. Sun, K. Xiu, Z. Wang, N. Hu, L. Zhao et al., Multifunctional wearable humidity and pressure sensors based on biocompatible graphene/bacterial cellulose bioaerogel for wireless monitoring and early warning of sleep apnea syndrome. Nano Energy **108**, 108215 (2023). <https://doi.org/10.1016/j.nanoen.2023.108215>
16. X.-H. Chen, Z. Tang, F.-M. Li, H.-Q. Li, S.-F. Li et al., Smart and low-cost flexible strain sensor based on graphene-MWCNT porous elastic sponge for home control and object grasping recognition using machine learning. ACS Appl. Electron. Mater. **7**(18), 8516–8527 (2025). <https://doi.org/10.1021/acsaelm.5c01275>
17. L. Wang, M. Zhang, B. Yang, J. Tan, X. Ding, Highly compressible, thermally stable, light-weight, and robust aramid nanofibers/Ti(3)AlC(2) MXene composite aerogel for sensitive pressure sensor. ACS Nano **14**(8), 10633–10647 (2020). <https://doi.org/10.1021/acsnano.0c04888>
18. Z. Wang, Z. Qin, B. Zhao, H. Zhu, K. Pan, Lightweight, superelastic, and temperature-resistant rGO/polysulfoneamide-based nanofiber composite aerogel for wearable piezoresistive sensors. J. Mater. Chem. C **11**(42), 14641–14651 (2023). <https://doi.org/10.1039/d3tc02496b>
19. G. Yang, X. Qin, T. Chen, J. Wang, L. Ma et al., Ultralight, superelastic pure graphene aerogel for piezoresistive sensing application. J. Mater. Sci. **58**(2), 850–863 (2023). <https://doi.org/10.1007/s10853-022-08113-8>
20. F. Niu, Z. Qin, L. Min, B. Zhao, Y. Lv et al., Ultralight and hyperelastic nanofiber-reinforced MXene–graphene aerogel for high-performance piezoresistive sensor. Adv. Mater. Technol. **6**(11), 2100394 (2021). <https://doi.org/10.1002/admt.202100394>
21. X. Zhang, Y. Wang, L. Zhang, X. Zhang, Y. Guo et al., Facile preparation of porous MXene/cellulose nanofiber composite for highly-sensitive flexible piezoresistive sensors in e-skin. Chem. Eng. J. **505**, 159369 (2025). <https://doi.org/10.1016/j.cej.2025.159369>
22. A. Li, J. Xu, S. Zhou, Z. Zhang, D. Cao et al., All-paper-based, flexible, and bio-degradable pressure sensor with high moisture tolerance and breathability through conformally surface coating. Adv. Funct. Mater. **34**(52), 2410762 (2024). <https://doi.org/10.1002/adfm.202410762>
23. Li, J. Xu, D. Xu, Z. Zhang, D. Cao et al., High-performance, breathable, and degradable fully cellulose-based sensor for multifunctional human activity monitoring. Chem. Eng. J. **505**, 159564 (2025). <https://doi.org/10.1016/j.cej.2025.159564>
24. Y. Cheng, M. Wang, N. Ma, R. Zhang, Z. Cai et al., Nanoscale interlayer engineering enhances MXene-based flexible pressure sensor. Nano Lett. **25**(31), 11782–11789 (2025). <https://doi.org/10.1021/acs.nanolett.5c01464>
25. M. Wang, G. Wang, M. Zheng, L. Liu, C. Xu et al., High-performance flexible piezoresistive pressure sensor based on multi-layer interlocking microstructures. J. Mater. Chem. A **12**(34), 22931–22944 (2024). <https://doi.org/10.1039/D4TA03758H>
26. H.-W. Zhang, X. Xu, D.-W. Jiang, J. Lu, Y.-S. Wang et al., MXene-integrated printed piezoresistive flexible sensors: a breakthrough in real-time monitoring for medical and smart applications. Adv. Sci. **12**(40), e10894 (2025). <https://doi.org/10.1002/advs.202510894>
27. X. Zhao, J. Yang, Y. Zhao, W. Zhai, K. Zhou et al., Flexible pressure sensor based on CNTs/CB/PDMS sponge with porous and microdome structures for sitting posture discrimination. Chem. Eng. J. **502**, 157878 (2024). <https://doi.org/10.1016/j.cej.2024.157878>
28. M. Wang, G. Wang, M. Zheng, W. Liu, W. Lv et al., High-performance flexible pressure sensor based on synergistic enhancement of magnetic field oriented carbon Nanotube/Graphene and microdome array structure. Chem. Eng. J. **511**, 162053 (2025). <https://doi.org/10.1016/j.cej.2025.162053>
